# Rewiring of Lipid Metabolism and Storage in Ovarian Cancer Cells after Anti-VEGF Therapy

**DOI:** 10.3390/cells8121601

**Published:** 2019-12-09

**Authors:** Matteo Curtarello, Martina Tognon, Carolina Venturoli, Micol Silic-Benussi, Angela Grassi, Martina Verza, Sonia Minuzzo, Marica Pinazza, Valentina Brillo, Giovanni Tosi, Ruggero Ferrazza, Graziano Guella, Egidio Iorio, Adrien Godfroid, Nor Eddine Sounni, Alberto Amadori, Stefano Indraccolo

**Affiliations:** 1 Veneto Institute of Oncology IOV-IRCCS, 35128 Padova, Italy; matteo.curtarello@iov.veneto.it (M.C.); martina.tognon@iov.veneto.it (M.T.); carolinaventuroli@live.it (C.V.); micol.silicbenussi@iov.veneto.it (M.S.-B.); angela.grassi@iov.veneto.it (A.G.); martina2.verza@gmail.com (M.V.); maricapinaz@alice.it (M.P.); albido@unipd.it (A.A.); 2 Department of Surgery, Oncology and Gastroenterology, University of Padova, 35128 Padova, Italy; soniaanna.minuzzo@unipd.it; 3 Department of Biology, University of Padova, 35128 Padova, Italy; valentina.brillo@studenti.unipd.it (V.B.); giovanni.tosi.1@studenti.unipd.it (G.T.); 4 Department of Physics, University of Trento, Via Sommarive 14, 38123 Trento, Italy; ruggero.ferrazza@yahoo.it (R.F.); graziano.guella@unitn.it (G.G.); 5 Core Facilities, NMR and MRI Unit, Istituto Superiore di Sanità, 00161 Roma, Italy; egidio.iorio@iss.it; 6 Laboratory of Tumor and Development Biology, GIGA-Cancer, University of Liège, 4000 Liège, Belgium; adrien.godfroid@ulg.ac.be (A.G.); nesounni@ulg.ac.be (N.E.S.)

**Keywords:** ovarian cancer, bevacizumab, metabolism, lipid droplets, LXR agonist

## Abstract

Anti-angiogenic therapy triggers metabolic alterations in experimental and human tumors, the best characterized being exacerbated glycolysis and lactate production. By using both Liquid Chromatography-Mass Spectrometry (LC-MS) and Nuclear Magnetic Resonance (NMR) analysis, we found that treatment of ovarian cancer xenografts with the anti-Vascular Endothelial Growth Factor (VEGF) neutralizing antibody bevacizumab caused marked alterations of the tumor lipidomic profile, including increased levels of triacylglycerols and reduced saturation of lipid chains. Moreover, transcriptome analysis uncovered up-regulation of pathways involved in lipid metabolism. These alterations were accompanied by increased accumulation of lipid droplets in tumors. This phenomenon was reproduced under hypoxic conditions in vitro, where it mainly depended from uptake of exogenous lipids and was counteracted by treatment with the Liver X Receptor (LXR)-agonist GW3965, which inhibited cancer cell viability selectively under reduced serum conditions. This multi-level analysis indicates alterations of lipid metabolism following anti-VEGF therapy in ovarian cancer xenografts and suggests that LXR-agonists might empower anti-tumor effects of bevacizumab.

## 1. Introduction

Tumors often exhibit altered metabolism compared with matched normal tissues of origin. Canonical alterations in metabolic pathways in tumor cells involve glycolysis, amino acid (especially glutamine, serine, and proline), and lipid metabolism [1]. Furthermore, recent studies uncovered therapy-induced changes in tumor metabolism. Accumulating evidence suggests that both chemotherapy and targeted therapies lead to a profound rewiring of tumor metabolism (reviewed in [2]); whether these metabolic adaptations contribute to resistance to therapy is a topic of increasing interest. Anti-angiogenic therapy is known to cause hypoxia and nutrient starvation both in experimental and clinical tumors, and it is thus considered a prototype of biological therapy able to exacerbate metabolic alterations in the solid tumor microenvironment [3]. Bevacizumab is an anti-VEGF monoclonal antibody which has been used also in the clinical management of advanced ovarian cancer patients, both at diagnosis and relapse [4,5]. Pre-clinical studies, including ours [6,7], have clearly shown that anti-VEGF therapy causes marked increase of glycolysis in ovarian cancer xenografts. Most of these metabolic perturbations are considered to occur as consequence of the increased hypoxia caused by anti-angiogenic therapy, although they are also found in untreated tumors, where they are generally limited to the hypoxic area of the mass [3]. Notably, not all of the metabolic changes caused by anti-VEGF therapy are due to hypoxia; most of them actually pre-exist and become selected in the nutrient poor and acidic cancer microenvironment. In fact, acidosis—which is common in hypoxic tumors and when vascular supply is limited [8]—triggers a recently described acidosis-induced metabolic phenotype consisting of simultaneous activation of fatty acid (FA) synthesis and FA oxidation supporting oxidative phosphorylation (OXPHOS) [9].

Although several studies reported on modulation of glycolysis and OXPHOS in tumors treated with anti-angiogenic drugs [7,10,11,12], much less is known with regard to perturbations of other metabolic pathways. Alterations in lipid metabolism have been reported by many studies, which collectively indicate that tumors often exhibit a lipogenic phenotype [13]. Part of these metabolic alterations have been attributed to the effects of hypoxia on lipid metabolism [14]. Specific examples include elevated rates of lipid synthesis accounted for by increased expression of various lipogenic enzymes, such as fatty acid synthase (FASN), which is strongly correlated with cancer progression [13,15]. In other models, increased FA uptake through FA binding proteins (FABPs) [16] or the FA channel protein CD36 [14] have been reported. With regard to alterations in the lipidomic profile after anti-angiogenic therapy, previous studies focused on what happens during the re-oxygenation phase following interruption of sunitinib, an anti-angiogenic tyrosine kinase inhibitor, and reported increased FA synthesis in several tumor models [17]. Moreover, with regard to bevacizumab, the most used anti-angiogenic drug in cancer patients, Bensaad et al. described increased lipid accumulation in tumor cells during bevacizumab treatment and under hypoxic conditions but in vivo data were exclusively based on the U87 glioblastoma model [16]. Therefore, whether published findings have broad significance is currently unknown. Considering the heterogeneous behavior of tumors following drug treatment, widening knowledge of the lipidomic changes occurring in the hypoxic microenvironment of tumors treated with anti-angiogenic drugs is important as it could eventually lead to identification of novel targets for combination therapies able to improve the rather limited state-of-the-art about efficacy of angiogenesis inhibition in ovarian cancer and other malignancies [18,19].

## 2. Materials and Methods

### 2.1. Cell Culture and Treatments

Established ovarian cancer cell lines, including IGROV-1, OC316, OVCAR3, and SKOV3 cells, were used in this study [7]. IGROV-1 cells were purchased from ATCC (Manassas, Virginia, USA). S. Canevari (INT; Milan, Italy, Europe) kindly provided OVCAR3 and SKOV3 cells. S. Ferrini (IST; Genoa, Italy, Europe) supplied OC316 cells. Authentication of specific genetic fingerprints by short tandem repeat (STR) DNA profile analysis showed that the cell lines presented exactly the same expected loci number profile, and confirmed their genetic identity (data not shown). 

IGROV-1, OC316, and SKOV3 cells were grown in RPMI 1640 (Euroclone; Pero, Italy, Europe) supplemented with 10% FBS (ThermoFisher Scientific; Waltham, Massachusetts, USA), 1% HEPES 10 mM (Cambrex Bioscience; Walkersville, Maryland, USA), 1% l-glutamine (2 mM), 1% Na pyruvate (1 mM) and 1% antibiotics-antimycotic mix (ThermoFisher Scientific; Waltham, Massachusetts, USA). OVCAR3 cells were grown in the same growth medium supplemented with 20% FBS. Cultures were maintained at 37 °C in a humidified 5% CO_2_/95% air atmosphere. Where specified, tumor cells were treated with certain compounds, including C75 (Adipogen AG; Liestal, Switzerland, Europe), or GW3965 (Selleckchem; Munich, Germany, Europe) before lipid droplets (LD) quantification and proliferation evaluation. In some experiments, oleic acid (Sigma Aldrich; St. Louis, Missouri, USA) was also used. Hypoxic treatment (0.5% O_2_) was achieved by incubating cells in an InvivO_2_ 300 hypoxic chamber (Ruskinn Technology; Pencoed, UK, Europe).

### 2.2. In Vivo Experiments

Procedures involving animals and their care were conformed to institutional guidelines that comply with national and international laws and policies (EEC Council Directive 86/609, OJ L 358, 12 December, 1987) and were authorized by the Italian Ministry of Health (authorization no. 617/2016-PR). For tumor establishment, eight-week-old nonobese diabetic/severe combined immunodeficiency (NOD/SCID) mice (Charles River; Wilmington, Massachusetts, USA) were subcutaneously (s.c.) injected into both flanks with 0.3−0.5 × 10^6^ tumor cells mixed at 4 °C with liquid Matrigel (BD; Franklin Lakes, NJ, USA). Tumor volume (mm^3^) was calculated as previously reported [20]. When tumors were about 150 mm^3^, anti-human VEGF mAb (bevacizumab) was administered i.p. at 100 µg/dose bi-or tri-weekly to NOD/SCID and mice were sacrificed 48 h after the last treatment. Control mice received i.p. injections of PBS.

### 2.3. Histology and Immunohistochemistry

Quantification of necrosis was carried out by calculating the percentage of the necrotic area in the entire tumor section, after staining with hematoxylin and eosin, by using a light microscope equipped with digital camera and MODEL software (Leica Microsystems; Wetzlar, Germany, Europe). For immunohistochemical analysis, 5 µm-thick paraffin-embedded tumor sections were re-hydrated and antigen retrieval was performed by incubation with citrate buffer 0.01 M pH 6.0 at 95 °C for 20 min. Then, slides were saturated with 5% pre-immune serum. To evaluate microvessel density (MVD), slides were incubated with rat anti-CD31 (1:50 dilution, cat. 550,274; BD Pharmingen; Franklin Lakes, NJ, USA), according to the manufacturer’s instructions. To investigate expression of phosphorylated AMPK kinase (pAMPK), slides were incubated with rabbit anti-pAMPK monoclonal antibody (1:100, Thr172, Cell Signaling Technology; Denvers, Massachusetts, USA). Citric acid buffer (pH 6.0, 10 mM) was used for antigen retrieval in all cases. IHC was performed using a Leica autostainer and the Bond Polymer Refine Detection kit (Leica Microsystems; Wetzlar, Germany, Europe). Immunostaining was visualized following substrate chromogen incubation: 3,3-Diaminobenzidine tetrahydrochloride hydrate (DAB) (10 min), followed by hematoxylin counterstaining (5 min). Positive control tissue samples were used as recommended by the manufacturer of the primary antibodies. Staining intensity and proportion were both considered in a scoring system utilized for these markers in our previous study [21]. Briefly, scores range from 0 to 18, based on the percentage of stained cells and on the intensity of staining. Intensity was given scores 0–3 (no staining = 0, light staining = 1, moderate staining = 2 and strong staining = 3) and the proportion was given scores 1–6 (0–4% = 1, 5–19% = 2, 20–39% = 3, 40–59% = 4, 60–79% = 5, 80–99% = 6).

### 2.4. Lipid Extraction and LC-MS Measurements

Lipids were extracted using a slight modification of the Bligh and Dyer protocol [22]: tumors were placed in a glass centrifuge tube, to which 3.75 mL of MeOH:CHCl_3_ 2:1 mixture was added. The samples were thoroughly mixed and mechanically disrupted by means of a homogenizer, while keeping the centrifuge tubes in a bath of water and ice. Twenty microliters (20 μL) of 1,2-dilauroyl-sn-glycero-3-phosphocholine (DLPC) 0.159 mg/mL standard solution was added to assess possible fluctuations in extraction yield. The samples were sonicated and vortexed for 15 min, then 1.25 mL CHCl_3_ was added, and the samples were sonicated and mixed again for another 15 min. A total of 1.25 mL H_2_O was added, and there followed a further sonication/mixing step. Finally, they were centrifuged for 15 min at 2000 rpm to induce phase separation. The organic (bottom) phase was recovered in a round-bottom flask, and the extraction procedure was repeated. The flask was then dried using a rotary evaporator and the lipids dissolved in a MeOH:CHCl_3_ 8:2 solution.

The LC-MS measurements were performed in both positive and negative ionization modes using a Waters Xevo G2 quadrupole time-of-flight (Q-ToF) combined with an Acquity UPLC system (Waters Corporation; Manchester, UK, Europe). Ten microliters (10 μL) of each sample was injected into an Acquity UPLC Charged Surface Hybrid (CSH) C18 column (1.7 μm × 2.1 mm × 100 mm, Waters Corporation; Manchester, UK, Europe) held at 55 °C. The flow rate was 0.4 mL/min, and the binary solvent system consisted of solvent A, HPLC-grade acetonitrile:water (60:40) with 10 mM ammonium formate, and solvent B, HPLC-grade acetonitrile:isopropanol (10:90) with 10 mM ammonium formate. The gradient elution program started from 40% B, reached 99% B in 18 min, then returned back to the starting condition, remaining there for 2 min. The MS data was collected over the *m*/*z* range of 100–1800 with a scan duration of 0.2 s. The source temperature was set at 120 °C and nitrogen (900 L/h) was used as the desolvation gas. The voltages of the sampling cone, extraction cone and capillary were 30 kV, 3.5 kV, and 2 kV, respectively, with a collision energy of 6 V for each full scan, and a collision ramp from 20 to 40 V for fragmentation. As lock mass, a solution of 2 ng/μL acetonitrile:water (50:50) leucine enkephalin (*m*/*z* 556.2771) with 0.1 % formic acid was infused into the instrument every 30 s.

### 2.5. NMR Tissue Metabolomics

Tissue samples were rapidly frozen in liquid nitrogen after collection to immediately stop any enzymatic or chemical reactions. Lipid and polar metabolites were extracted using the dual phase extraction method [23]. Briefly, tissues were homogenized and extracted with ice cold methanol/chloroform/water (1:1:1) and vigorously vortexed. Samples were stored at 4 °C overnight. After phase separation by centrifugation at 20,000× *g* at 4 °C for 30 min; the polar water-methanol phase containing water soluble cellular metabolites methanol was evaporated using a rotary evaporator and then lyophilized; while the organic phase (lipid phase) was collected in the tube and chloroform was evaporated under nitrogen gas. Both phases of cell extracts were stored at −20 °C. High-resolution 1H NMR analyses were performed at 25 °C at 400 MHz (9.4 T Bruker AVANCE spectrometer; Karlsruhe, Germany, Europe) on aqueous and organic cell extracts using acquisition pulses, water pre-saturation, data processing, and peak area deconvolution as previously described [24,25]. Quantification of individual metabolites was obtained from peak areas applying the correction factors determined by experiments at equilibrium of magnetization ([90]° pulses, 30.00-s inter-pulse delay). Metabolite quantification was expressed as metabolite percentage relative to total metabolites. All data were calculated as mean ± SD.

### 2.6. Lipid Droplets (LD) Evaluation

Tumor sections were labelled with rabbit anti-human adipophilin (ADRP) mAb (1:500 dilution; cat. 52,355; Abcam; Cambridge Science Park, UK, Europe) followed by staining with a monkey anti-rabbit 555 secondary antibody (Invitrogen, Milan, Italy, Europe), and quantification was performed on whole tumor sections from five to six different tumors using computerized quantification of ADRP-positive cells divided by DAPI-positive cells. Staining density was calculated by manual exclusion of necrotic areas. Data acquisition was performed by using MATLAB software as described elsewhere [17]. Nuclei were stained with DAPI (Invitrogen; Milan, Italy, Europe).

Fluorescent dye 4,4-difluoro-1,3,5,7,8-pentamethyl-4-bora-3a,4a-diaza-s-indacene (5 mM BODIPY 493/503 dye) (D3922; ThermoFisher Scientific; Waltham, Massachusetts, USA), which binds intracellular neutral lipids, was also utilized to evaluate LD in vitro. A total of 3.0 × 10^5^−5.0 × 10^5^ cells were incubated on BODIPY staining solution (BODIPY diluited 1:2500 in PBS) in the dark for 15 min at 37 °C. After washing with PBS, cells were re-suspended in 300 L of 1X flow cytometry buffer (0.01 M HEPES (pH 7.4), 0.14 M NaCl, 2.5 mM CaCl_2_) and analyzed on a LSR II cytofluorimeter (BD; Franklin Lakes, NJ, USA). In some experiments, cell samples were analyzed on a Zeiss LSM 510 microscope (Zeiss, Jena, Germany) and LD were quantified as number of pixels for field. In a set of experiments, CD117^+^ CSCs were measured by flow cytometry in cell cultures freshly established from tumor xenografts or in cell lines grown under normoxic or hypoxic conditions. To this end, 3.0 × 10^5^−5.0 × 10^5^ cells were incubated with APC-mouse anti-human CD117 antibody (BD Biosciences; Allschwil, Switzerland, Europe), diluted 1:1000 in PBS, in the dark for 15 min at 37 °C. After washing with PBS, cells were re-suspended in 300 µL of 1X flow cytometry buffer (0.01M HEPES (pH 7.4), 0.14 M NaCl, 2.5 mM CaCl_2_) and analyzed on a LSR II cytofluorimeter (BD; Franklin Lakes, NJ, USA).

### 2.7. Proliferation Assay

Proliferation, after incubation with GW3965 in normoxic and/or hypoxic conditions, was measured by the CellTiter96^®^ AQueous One Solution Cell Proliferation Assay (Promega; Madison, WI, USA).

### 2.8. Annexin-V Apoptosis Assay

Cell viability was evaluated using Annexin V/PI Staining Kit (Roche Applied Sciences; Penzberg, Germany, Europe). Cells were stained with 2 μL Annexin-V Fluos, 2 μL propidium iodide, and 100 μL HEPES buffer, according to the manufacturer’s instruction. Following an incubation of 15 min in the dark, staining was blocked with 200 μL HEPES buffer. Labelled cells were analyzed by an LSR II cytofluorimeter (BD; Franklin Lakes, NJ, USA).

### 2.9. Microarray Expression Analysis

RNA quality and purity control was assessed with the Agilent Bioanalyzer 2100 (Agilent Technologies; Waldbronn, Germany, Europe) and a eukaryote total RNA nano assay (Agilent). For microarray expression experiments, only the total RNA of high quality was used (RIN > 7). RNA samples that passed the high-quality controls were diluted to 100 ng in a total volume of 3 μL DEPC-treated water. In vitro transcription and biotin labelling were performed according to the GeneChip 3’IVT Express kit protocol (Affymetrix; Santa Clara, CA, USA). Following fragmentation, biotinylated cRNA was hybridized for 16 h at 45 °C to GeneChip™ PrimeView™ Human Gene Expression Arrays in an Affymetrix GeneChip hybridization oven 645. Affymetrix Fluidics Station 450 was used to stain and wash the chips. Arrays were then scanned on an Affymetrix GeneChip Scanner GCS3000 and the image (*.DAT) files were processed using the Affymetrix GeneChip Command Console (AGCC) software v.5.0 to generate cell intensity (*.CEL) files. Prior to transcriptional data analysis, a chip quality assessment was executed using the Affymetrix Expression Console software v.1.4 and for every array all quality metrics were found within boundaries. Bioinformatic analysis was carried out in the R statistical environment using the Bioconductor package [26]. Data were preprocessed using the RMA algorithm [27]. Differential expression analysis was performed using the limma package, by linear modelling, moderating the t-statistics by empirical Bayes shrinkage [28]. To correct for multiple testing, the Benjamini and Hochberg’s method was applied.

Gene set enrichment analysis (GSEA) was performed to evaluate the functional significance of curated sets of genes [29]. Genes were ranked by moderated t-statistics and GSEA pre-ranked was run with default parameters against the Reactome canonical pathways present in the “c2.cp.reactome” collection of the Molecular Signatures Database v5.2 (http://www.broadinstitute.org/gsea/msigdb/index.jsp). Gene sets with a nominal *p*-value < 0.02 and a false discovery rate FDR < 0.25 were defined as significantly enriched.

Microarray data, together with the description of experiments, protocols and results of differential expression analysis, have been deposited in the ArrayExpress database (www.ebi.ac.uk/arrayexpress) under accession number E-MTAB-7683.

### 2.10. Western Immunoblotting

For Western blot analysis, IGROV-1 and SKOV3 cells were lysed on ice in a RIPA lysis buffer (Cell Signaling Technology; Denvers, Massachusetts, USA) in the presence of 1× phosphatase (Calbiochem; San Diego, CA, USA) and protease inhibitors (Sigma Aldrich; St. Louis, Missouri, USA). Lysates were then cleared with a centrifugation at 13,000× *g* for 30 min at 4 °C, and proteins were quantified using a quantum protein assay (Euroclone; Pero, Italy, Europe). About 30 μg of proteins were denatured and loaded in a midi polyacrylamide gel 4–12% (ThermoFisher Scientific; Waltham, Massachusetts, USA). Separated proteins were then transferred for 2 h at 400 mA on a nitrocellulose membrane (GE Health Care, Glattbrugg, Switzerland, Europe). Membranes were saturated for 1 h with TBS-0,1% Tween-5%-milk and then incubated over night with primary antibodies at 4 °C, according to manufacturer’s instructions.

Primary antibodies used:

- Fatty Acid Synthase (FAS) (C20G5) #3180, 1:1000 (Cell Signaling Technology; Denvers, Massachusetts, USA)
- CD36/SR-B3 (CD36) NB400-144, 1:500 (NOVUSBIO; Minneapolis, Minnesota, USA)

Membranes were washed twice for 15 min and incubated a 1:5000 diluted HRP-conjugated anti-mouse or anti-rabbit secondary antibodies (Amersham-Pharmacia; Little Chalfont, UK, Europe). Detection was obtained using Western Lightning plus ECL reagents (PerkinElmer; Waltham, Massachusetts, USA), containing Luminol, which is oxidized by horseradish peroxidase, resulting in light emission at 425 nm. Signals emitted were acquired using a UVITEC imaging system (UVITEC; Cambridge, UK, Europe).

### 2.11. Statistical Analysis

Results were expressed as mean value ± SD. Statistical comparison between two sets of data was performed using the unpaired Student’s *t* test (two-tailed). Differences were considered statistically significant with *p* < 0.05 (*). For ADP staining quantification, considering that the distributions are non-Gaussian, a non-parametric one-tailed Mann–Whitney test was applied.

## 3. Results

### 3.1. Anti-VEGF Therapy Causes Heterogeneous, Model-Dependent Effects on the Tumor Lipidome

**Figure 1:**
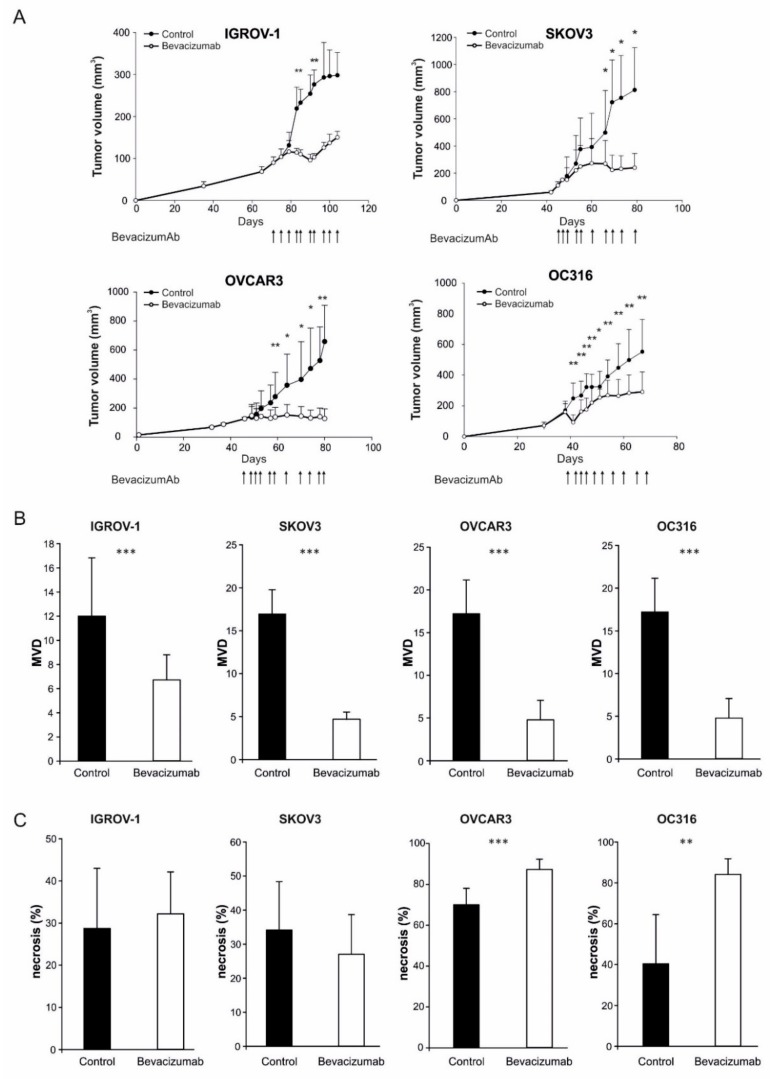
Effects of anti-VEGF therapy on tumor growth, microvessels density and necrosis. (**A**) Kinetics of tumor development in NOD/SCID mice s.c. injected with ovarian cancer cell lines (IGROV-1, SKOV3, OVCAR3, and OC316) and the effects of multiple injections of the anti-VEGF mAb bevacizumab (arrows, 100 µg/dose, administered bi-or three-weekly) on tumor size compared to controls (*n* = 6 mice for group), * *p* < 0.05, ** *p* < 0.01, *t*-test. Tumors were collected and analyzed two days after the last dose of anti-VEGF mAb or PBS for bevacizumab and control groups, respectively. (**B**) Microvessels density (MVD) evaluation by staining with anti-CD31 mAb. Columns show mean ± SD values (*n* = 5–10 fields for tumor; *n* = 6 tumors for group), *** *p* < 0.001, *t*-test. (**C**) Columns indicate quantitative analysis of necrotic areas in *n* = 6 different tumors for each group, ** *p* < 0.01, *** *p* < 0.001, *t*-test.

In the initial part of this study, we established subcutaneous xenografts of four human ovarian cancer cell lines in NOD/SCID mice and treated them with bi-weekly injections of the anti-VEGF antibody bevacizumab, as detailed under Materials and Methods. Significant delays in tumor growth were measured in each of these models (Figure 1A). Analysis of tumor samples obtained at time of sacrifice of the control mice indicated that these anti-tumor effects were associated, as expected, with a significant reduction of microvessel density (Figure 1B), underscoring angiogenesis inhibition. Tumor necrosis was markedly increased by bevacizumab in the OC316 and OVCAR-3 tumor models but not in the IGROV-1 and SKOV3 models (Figure 1C), in line with our previous data [7]. Phosphorylated AMPK (Thr172), a marker of the metabolic stress induced by bevacizumab [7], was clearly detected in IGROV-1 tumors, whereas it was marginally found in SKOV3 tumors (Figure S1), suggesting different levels of bevacizumab-induced AMPK activation.

**Figure 2:**
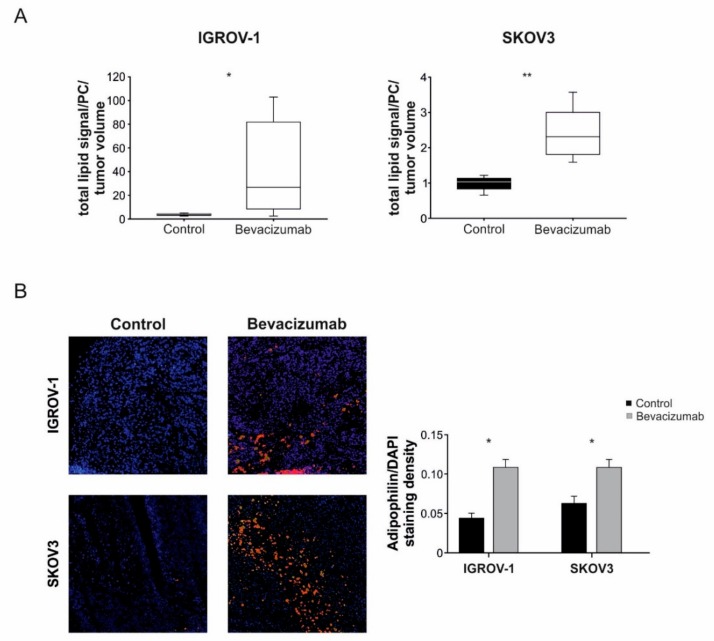
Increase of intra-tumor lipid amount following anti-VEGF therapy. (**A**) Evaluation of the tumor lipidome component by LC-MS in IGROV-1 and SKOV3 tumor xenografts following anti-VEGF therapy. Total lipids signal is normalized to 1,2-dilauroyl-sn-glycero-3-phosphocoline (DLPC) signal and to tumor volume (mm^3^). Columns show mean ± SD values (*n* = 6 tumors for group), * *p* < 0.05, ** *p* < 0.01, *t*-test. (**B**) Representative images of immunofluorescence staining for adipophilin (ADRP) protein, a specific marker of lipid accumulation, in control and bevacizumab-treated tumors (**left**). Quantification of ADRP expression normalized to DAPI staining density. Columns show mean ± SD values (*n* = 6 tumors/group) * *p* < 0.05, Mann–Whitney test, (**right**).

We subsequently extracted lipids from freshly frozen tumor samples and analyzed them by LC-MS, focusing on IGROV-1 and SKOV3 models because these tumors were less necrotic compared with OC316 and OVCAR-3 tumors. The results, shown in Figure 2A, indicated that the overall amount of lipids normalized per tumor volume increased following bevacizumab administration with statistically significant modulations in both models. Prompted by these results, and in light of previous work showing increased amounts of lipid droplets (LD) in subcutaneous experimental tumors treated with anti-angiogenic drugs [16], we investigated lipid storage in our samples by staining with the LD-associated marker ADRP [30]. The results show increased numbers of ADRP^+^ cells in tumors treated with anti-VEGF therapy; this phenomenon was clearly observed in both tumor models analyzed (Figure 2B) and also in the thin viable tumor rim around necrosis of OVCAR3 and OC316 xenografts (data not shown).

**Figure 3:**
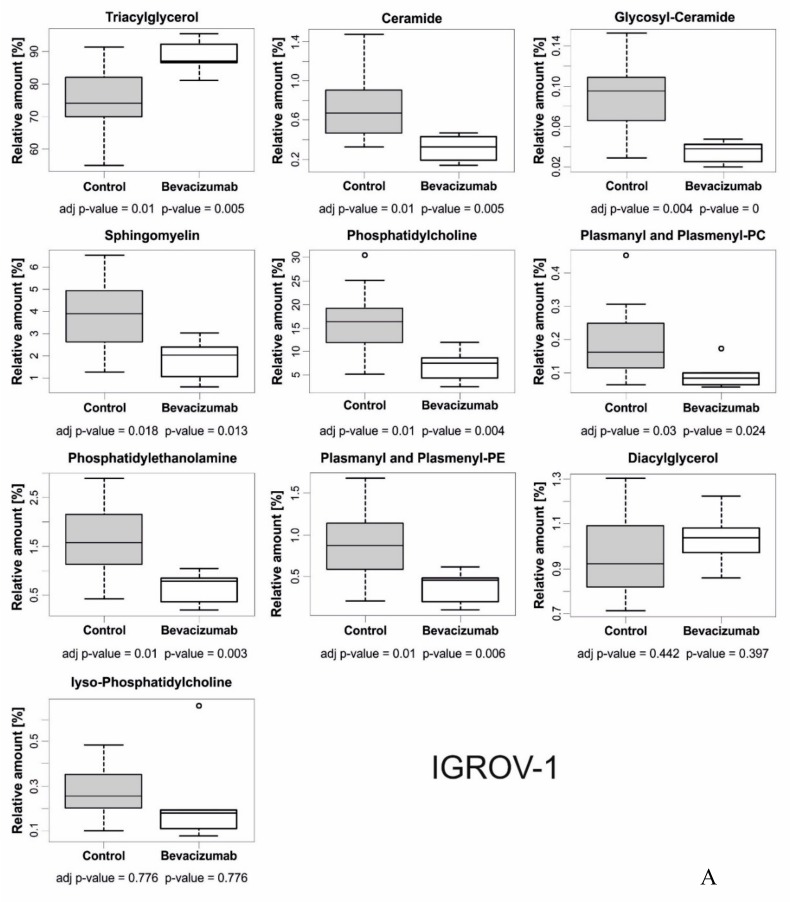

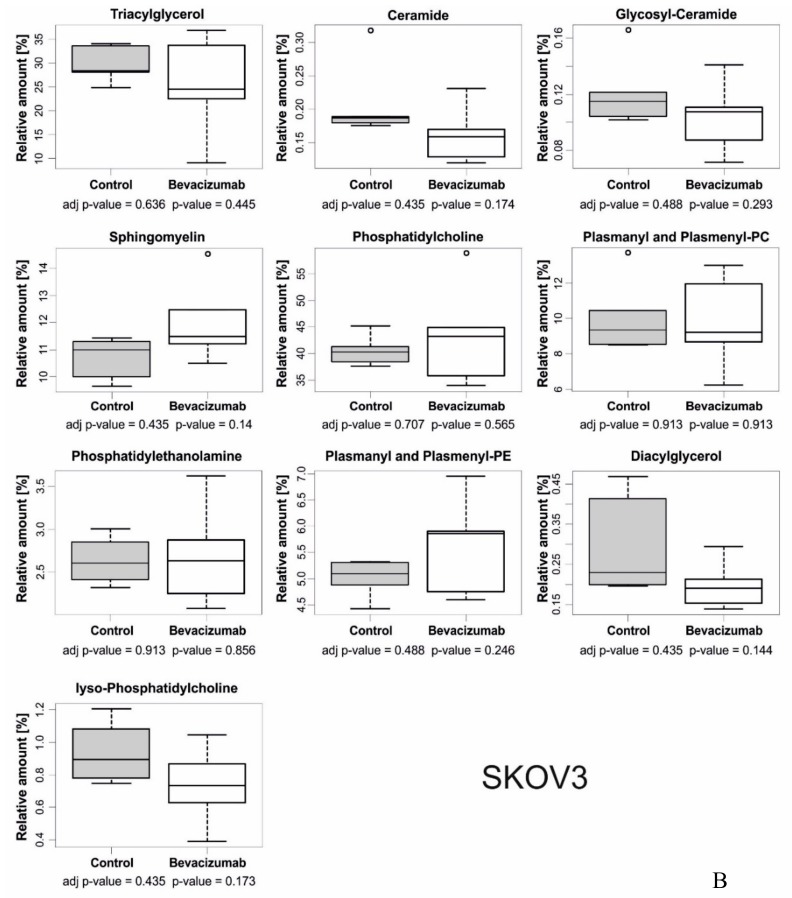
Relative abundances of different lipid classes in bevacizumab-treated and control tumors. (**A**) Evaluation of relative amounts of lipid classes in bevacizumab-treated IGROV-1 tumors compared to the control. Dot blots show mean ± SD values (6 tumors/group). Most of the results are statistically significant (*adj p*-value < 0.05). (**B**) The same analysis showed only marginal differences between bevacizumab-treated and control tumors in the SKOV3 model.

We observed several model-specific changes in the relative amounts of different lipid classes and the degree of unsaturation. In the IGROV-1 model, triacylglycerol (TAG) was significantly increased in bevacizumab-treated tumors compared with control tumors (Figure 3A), whereas several other lipid classes, including ceramide, glycosyl ceramide, sphingomyelin, phosphatidylcholine, plasmanyl and plasmenyl-PC, phosphatidylethanolamine, and plasmanyl and plasmenyl-PE, were relatively reduced in bevacizumab-treated tumors compared with control tumors (Figure 3A). In contrast, we did not find any significant difference in lipid classes in SKOV3 tumors (Figure 3B), despite increased LD accumulation (Figure 2B). With regard to variations in chain length, unsaturations, and unsaturation index, considering both tumor models and the different lipid classes, we observed a trend towards shorter lipid chains and increased unsaturations (Table S1).

To confirm and broaden these findings, we analyzed the lipid content of tumors by both MS and NMR approaches, which disclosed increased TAG, total phospholipids and polyunsaturated chains in bevacizumab-treated compared with control IGROV-1 tumors (Figure S2), whereas these alterations were not detected in SKOV3 tumors (Figure S3).

Altogether, these findings indicate that although anti-VEGF therapy was followed by increased lipid accumulation in both tumor models, marked qualitative alterations of the lipidome were detected by LC-MS and both MS and NMR analyses predominantly in the IGROV-1 model.

### 3.2. Anti-VEGF Therapy is Associated with Increased Expression of Components of TAG and Glycerophospholipid Biosynthesis

We investigated possible mechanisms accounting for increased lipid storage in tumors treated with anti-VEGF therapy. Transcriptome analysis disclosed heterogeneous results in the models analysed. In fact, several pathways linked to metabolism, including mainly glycolysis and lipid metabolism, were found up-regulated in IGROV-1 tumors treated with the anti-VEGF antibody compared with controls (Table S2), whereas other pathways linked to biosynthesis of components of the extracellular matrix and glycosaminoglycans prevailed in SKOV3 tumors (Table S2). Notably, TAG and glycerophospholipid biosynthesis were among up-regulated pathways in IGROV-1 tumors, in line with results of MS and NMR analyses. Signaling by Notch was one of the few pathways up-regulated in both models (Table S2). With regard to down-regulated pathways, anti-angiogenic therapy was associated with reduced DNA replication and synthesis, DNA repair, respiratory electron transport, and apoptosis (Table S3).

We conclude that although certain transcriptional effects of bevacizumab treatment are shared by both tumor xenograft models, marked rewiring of pathways associated with lipid metabolism at the mRNA level is only found in the IGROV-1 tumor model.

### 3.3. Hypoxia Accounts for Increased LD Accumulation and Expression of Cancer Stem Cells (CSCs) Markers in Tumor Cells

**Figure 4:**
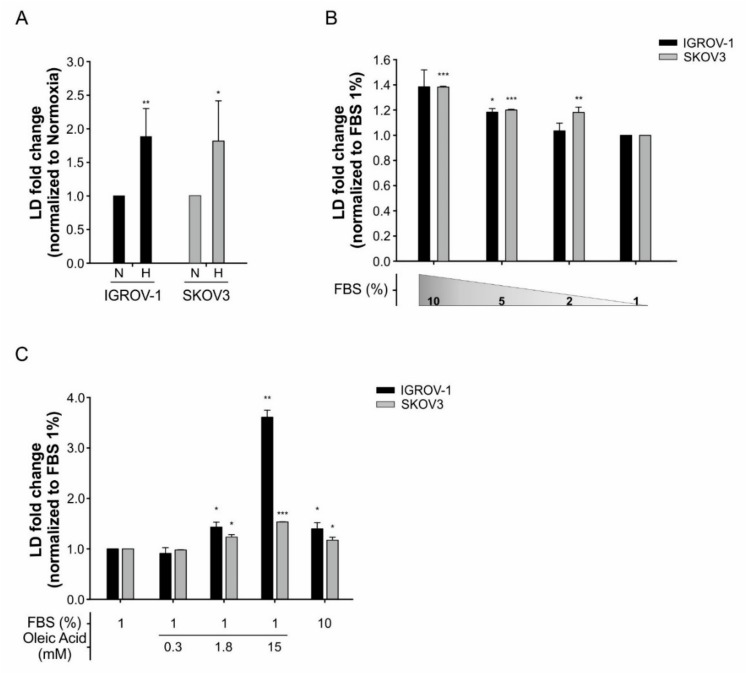
LD accumulation under hypoxia condition and serum starvation in IGROV-1 and SKOV3 ovarian cancer cells. (**A**) Quantification of LD in cancer cells, cultured under normoxia (N) or hypoxia (H) for 48 h, by flow cytometry analysis following staining with BODIPY 493/503 dye. X-mean values are normalized to normoxia condition. Columns show mean ± SD values of three experimental replicates (* *p* < 0.05, ** *p* < 0.01, *t*-test) (**B**) LD content in cancer cells cultured under normoxia and serum starvation for 24 h. X-mean values are normalized to 1% FBS condition. Columns show mean ± SD values of three experimental replicates (* *p* < 0.05, ** *p* < 0.01, *** *p* < 0.001, *t*-test). (**C**) LD content in cancer cells cultured under normoxia and 1% FBS condition with supplementation of oleic acid in three different concentrations (0.3 mM, 1.8 mM and 15 mM) for 24 h. Columns show mean ± SD values of three experimental replicates (* *p* < 0.05, ** *p* < 0.01, *** *p* < 0.001, *t*-test).

As hypoxia has been shown to account for increased LD formation in tumor cells [16] and anti-VEGF-treated IGROV-1 tumors have increased hypoxic areas [6], we investigated whether hypoxia could drive LD formation in the tumor cells used in this study. To this end, we incubated IGROV-1 cells under hypoxic conditions (0.5% O_2_) in vitro, labelled them with the LD marker BODIPY 493/503 dye and performed flow cytometry analysis. As expected, BODIPY^+^ cells significantly increased under hypoxia (Figure 4A), indicating that hypoxia contributes to increase LD formation. Similar results were obtained in SKOV3 cells (Figure 4A) and were confirmed by quantification of BODIPY^+^ cells by confocal microscopy analysis (Figure S4). We also observed that the increase in BODIPY^+^ cells was serum-dependent. In fact, lowering the percentage of FBS in medium from 10% to 1% under hypoxic conditions reduced BODIPY^+^ both in IGROV-1 and SKOV3 cells (Figure 4B). Finally, this phenomenon was counteracted by oleic acid (Figure 4C), suggesting that FBS provided exogenous lipids for uptake into tumor cells.

Previous studies indicated that increased LD levels are a feature of CSCs in ovarian cancer [31] as well as liver cancer [32]. We, therefore, investigated whether bevacizumab treatment or incubation under hypoxic conditions could increase CD117^+^ cells, a sub-population endowed with CSCs features in ovarian cancer [33]. We found that bevacizumab treatment is associated with increased levels of CD117^+^ cells in IGROV-1 tumors (Figure S5). Moreover, incubation under hypoxic conditions increased CD117^+^ CSCs in vitro, both in the IGROV-1 and SKOW3 models (Figure S5), as well as LD, although we did not find higher accumulation of LD in CSCs compared with non-CSC cells. We conclude that LD accumulation overlaps with selection by hypoxia or other effects of bevacizumab of cancer cells enriched for CSC markers.

### 3.4. LD Accumulation in Tumor Cells is Counteracted by the LXR-Agonist GW3965

Next, we investigated whether it might be possible to counteract the LD accumulation associated with incubation of tumor cells under hypoxia in vitro. LD are cytoplasmic organelles responsible for the storage of both cholesteryl esters and TAG [30]. In view of the increased amounts of lipids in tumors treated with bevacizumab, we focused on GW3965, a Liver X Receptor (LXR) agonist [34]. LXRs are members of the nuclear receptor transcription factor family and are critical transcriptional regulators of cholesterol metabolism, controlling cholesterol flow into cells and its catabolism [35]. Activation of the LXR system has been reported to affect lipid metabolic networks, to stimulate cholesterol efflux from the cells and inhibit proliferation of various types of tumor cells [34].

**Figure 5:**
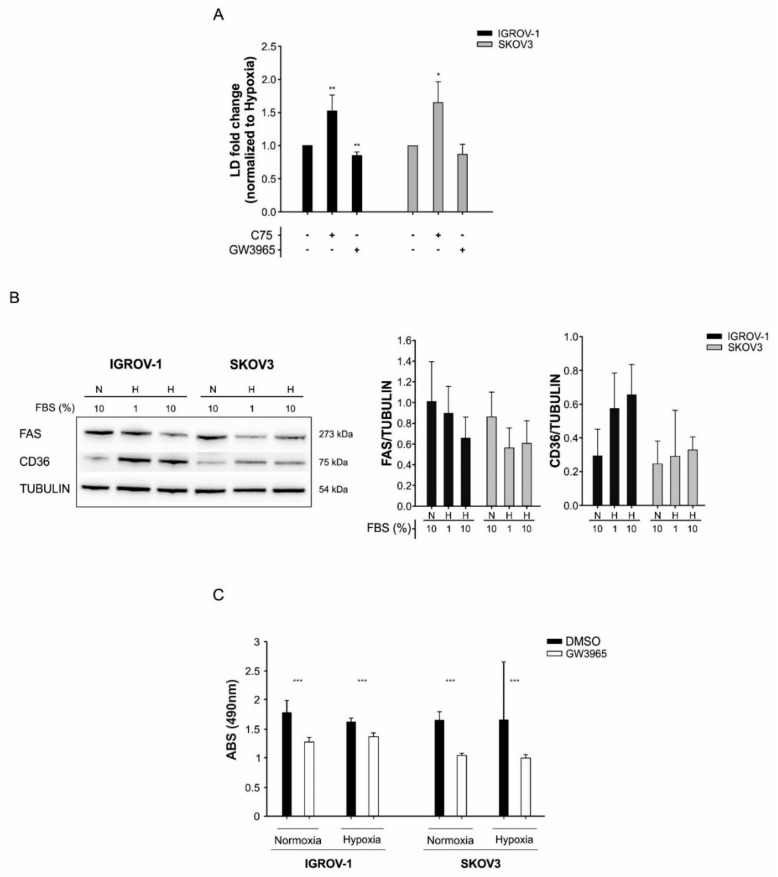
LD content and cell viability modulation by lipid metabolism’s inhibitors in IGROV-1 and SKOV3 ovarian cancer cells. (**A**) Evaluation of LD content in cancer cells cultured in hypoxia for 48h following treatment with the FASN inhibitor C75 and the LXR agonist GW3965 used at IC50 concentrations (C75 IC50 46 µM and 50 µM for IGROV-1 and SKOV3, respectively. GW3965 IC50 22.5 µM and 36.5 µM for IGROV-1 and SKOV3, respectively). X-mean values are normalized to hypoxia values. Columns show mean ± SD values of three experimental replicates (* *p* < 0.05, ** *p* < 0.01, *t*-test). (**B**) Effects of hypoxia and serum starvation on the expression of FAS and CD36 in ovarian cancer cells. Left panel: Western Blot analysis of FAS and CD36 expression in one representative experiment. Tubulin was used as loading control. Right panel: quantitative analysis of FAS and CD36 expression. Columns show mean ± SD values of three experimental replicates. N = normoxia, H = hypoxia. (**C**) GW3965 treatment decreased proliferation of tumor cells both under normoxia and hypoxia conditions. Columns show mean ± SD values of three experimental replicates (*** *p* < 0.001, *t*-test).

We initially determined the IC50 of GW3965 in IGROV-1 and SKOV3 cells with this drug, which was 22.5 µM and 36.5 µM, respectively. We measured a slight, but significant, reduction of BODIPY 493/503 uptake following GW3965 treatment under hypoxic conditions (Figure 5A). In contrast, the FASN inhibitor C75 [36] increased LD levels in tumor cells (Figure 5A). These in vitro findings were associated with upregulation of the membrane lipid transporter CD36 and reduction of FAS expression under hypoxic conditions (Figure 5B).

**Figure 6:**
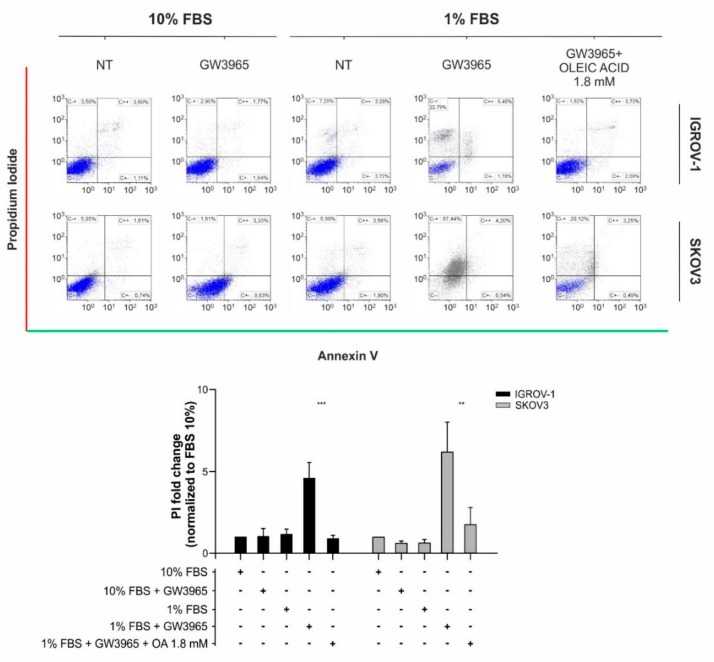
GW3965 effect on IGROV-1 and SKOV3 ovarian cancer cell death. Apoptosis/necrosis evaluation in cancer cells following GW3965 treatment at IC50 concentrations and under serum starvation (1% FBS) with supplementation of oleic acid (1.8 mM). Top panel: representative dot blots of cell populations with the *X*-axis for Annexin V positivity and the *Y*-axis for propidium iodide (PI) positivity. Bottom panel: Quantitative evaluation of necrosis. Data are normalized to PI positivity cells percentage under normal serum condition (10% FBS). Columns report the mean values ± SD of four different replicates for each condition (** *p* < 0.01, *** *p* < 0.001, *t*-test).

As expected, cell proliferation was inhibited by GW3965 treatment, both under normoxic and hypoxic conditions (Figure 5C), in line with previous findings [37]. Finally, although GW3965 had marginal cytotoxic effects under standard cell culture conditions, reduction of FBS to 1% was followed by massive cell death in vitro, which was partially rescued by supplementation of oleic acid (Figure 6, Figure S6). In conclusion, since anti-angiogenic therapy causes hypoxia and nutrient starvation in tumors, our findings suggest that LXR agonists might reduce lipid accumulation and cell proliferation in tumors treated with bevacizumab.

## 4. Discussion

Anti-angiogenic therapy deprives tumors of oxygen and nutrients and tends to exacerbate certain distortions of the metabolism commonly detected in the central and hypoxic areas of solid tumors [3]. In contrast to the large body of studies addressing alterations of lipid metabolism in tumors, to our knowledge only two previous studies investigated how anti-angiogenic therapy perturbs lipid metabolism in tumor models [16,17]. Sounni et al. reported that treatment of breast cancer xenografts with antiangiogenic TKI including sunitinib and sorafenib was associated with hypoxia and enhanced glycolysis, but when drug administration was stopped angiogenesis restoration and enhanced lipid synthesis were observed. Interestingly, pharmacological lipogenesis inhibition with the FASN inhibitor orlistat impaired tumor re-growth and metastasis after sunitinib treatment withdrawal [17]. Along this line, Bensaad et al. showed that treatment of the U87 glioblastoma xenograft model with bevacizumab was associated with FA uptake and lipid droplet accumulation induced by HIF-1α, while de novo FA synthesis was repressed in hypoxia. Moreover, inhibition of lipid storage by FABP3 or FABP7 knockdown decreased tumor cell survival under hypoxia-reoxygenation and impaired tumorigenesis in vivo [16]. Importantly, both studies showed that pharmacologic interventions targeting lipid synthesis or uptake could empower anti-tumor effects of anti-angiogenic therapy, although this concept has not been explored in additional studies. Inspired by these considerations, we investigated alterations in lipid metabolism by a multi-modal approach in ovarian cancer xenografts treated with bevacizumab. We exploited tumor xenograft models well characterized in terms of their response to bevacizumab and metabolic alterations triggered by anti-angiogenic treatment [6,7]. Our results indicate some shared and some heterogeneous responses in the models analyzed. In fact, although increased lipid content and LD accumulation were consistently observed, dysregulation of lipid metabolism at translational and post-translational levels was a feature only of the IGROV-1 model. Metabolomics analysis indicated an increased accumulation of TAG, as confirmed by both MS and NMR studies. We also observed a trend towards shorter lipid chains and increased unsaturations, partially in line with others reported by incubating cancer cells under hypoxia in vitro [38]. It is important to consider that mammalian cells have a limited ability to synthesize polyunsaturated FA de novo, as they lack the Δ12 desaturase [15]. Therefore, MS and NMR results suggest increased lipid uptake more than enhanced lipid synthesis in our tumor models. Transcriptomic changes also supported rewiring of lipid metabolism in the IGROV-1 model, although up-regulation of FABP3 and FABP7, reported by Bensaad et al. in U87 tumor xenografts treated with bevacizumab [16], was not found in our tumor models (data not shown). In contrast, minor changes were found in SKOV3 tumors, although anti-VEGF therapy exerted similar anti-tumor and anti-vascular effects. The reason behind this difference is unknown. Intriguingly, IHC staining disclosed that bevacizumab administration was followed by robust AMPK activation in IGROV-1 tumors, as we previously reported [7], but not in SKOV3 tumors where some levels of pAMPK expression were indeed detected but they did not increase following bevacizumab administration (Figure S1). AMPK controls cellular lipid metabolism through direct phosphorylation of ACC1 and ACC2, suppressing FA synthesis and simultaneously promoting FA oxidation by relieving the suppression of CPT1 [39]. AMPK also phosphorylates and inhibits HMGCR, which leads to reprogramming of lipid and sterol synthesis in cells and promotes lipid absorption through CD36. We speculate that differences in AMPK activation in vivo might account for the stronger modulation of lipid metabolism in IGROV-1 compared with the SKOV3 model, although we did not further investigate this in this study.

In vitro studies indicated that hypoxia increased lipid accumulation in tumor cells, and this phenomenon depended at least in part from increased uptake of exogenous lipids and was associated with increased AMPK activation and expression of the lipid importer CD36 (data not shown). Importantly, reduction of the percentage of FBS in medium prevented LD accumulation under hypoxia and this phenomenon was counteracted by adding oleic acid to the cells, showing the dependence of tumor cells from exogenous lipids under oxygen starvation. Moreover, treatment of tumor cells with the LXR agonist GW3965 counteracted LD accumulation, whereas a FASN inhibitor increased LD levels. Altogether, these findings remark the important biological contribution of lipid uptake to this phenomenon, which could also be in part driven by HIF-1 and HIF-2, which under hypoxic conditions repress CPT1A reducing FA transport into the mitochondria and forcing FA to LD for storage [40]. On the other hand, we are aware of the fact that in vitro growth culture conditions might only in part mimic the complexity of the tumor microenvironment and there are examples—albeit not related to lipid metabolism—showing that certain metabolic pathways preferred by tumor cells in vitro are barely utilized in the context of solid tumors [41]. Therefore, additional studies will be required to quantify the relative contributions of exogenous lipid uptake versus endogenous lipid synthesis in tumors treated with bevacizumab.

What is the biological relevance of lipid accumulation in ovarian cancer cells treated with bevacizumab? On one hand, rapid accumulation of cytoplasmic lipid droplets is a feature of apoptosis [42], and our findings could in part reflect induction of apoptosis by bevacizumab, in line with the results of transcriptome analysis. On the other hand, hypoxia-driven accumulation of LD could serve as a protective barrier against oxidative stress-induced toxicity [16]. LD are dynamic organelles that either store excess lipids or fuel cells with essential lipids to sustain lipid homeostasis. They are composed of a neutral lipid core (TAG and sterol-esters) surrounded by a phospholipid monolayer composed of PC and several proteins involved in lipid metabolism [43]. Triglycerides contained in LD have been reported to promote lipid homeostasis during hypoxic stress in kidney cancer [14]. Moreover, Qiu et al. published in clear cell renal tumors that lipid deposits offer protection from hypoxia or pharmacologically-induced ER stress [44]. Along this line, LD accumulation could drive resistance to drugs, such as 5-fluorouracil and oxaliplatin [45], which are often used in combination with bevacizumab in metastatic colorectal cancer, and there are emerging mechanisms involving adypocytes and lipid metabolism in altering the response to cancer treatment [46]. Finally, other studies established that increased LD are a hallmark of ovarian CSCs as well as liver CSCs [31,32]. Interestingly, in our models we also found increased expression of CD117, a marker of ovarian CSCs [33] both in *ex vivo* cultures from bevacizumab-treated tumors and in IGROV-1 and SKOV3 cells grown under hypoxic conditions. This finding indicates that LD accumulates in CSCs, thus supporting the development of novel drugs to counteract this phenomenon.

In view of the putative protective role of TAG and LD in tumors, our results indicating that drug-mediated modulation of the LXR system dramatically increased cell death especially under FBS starvation could be of translational relevance. Other studies previously reported that FBS can protect tumor cells from certain metabolic vulnerabilities [14]. Although LXR agonists have anti-tumor activity in other tumor models (reviewed in [35]) this is the first study to show that GW3965 empowers effects of hypoxia and serum starvation. Despite the fact that liver toxicity and other unforeseen adverse reactions have so far delayed clinical applications of LXR agonists [47], our observations suggest that LXR agonists might disclose stronger anti-tumor effects when combined with drugs which induce nutrient starvation, such as bevacizumab.

## Supplementary Materials

The following are available online at https://www.mdpi.com/2073-4409/8/12/1601/s1. Figure S1. AMPK activation in ovarian cancer xenografts following anti-VEGF therapy; Figure S2. Lipid content evaluation in IGROV-1-treated tumors compared to controls by NMR analysis; Figure S3. Lipid content evaluation in SKOV3-treated tumors compared to controls by NMR analysis; Figure S4. LD accumulation under hypoxic conditions in ovarian cancer cells; Figure S5. Analysis of CSCs in IGROV-1 tumors and under hypoxic conditions; Figure S6. Flow cytometric morphologic evaluation of ovarian cancer cells; Table S1. Summary of the lipid intra-class comparisons in bevacizumab versus control samples; Table S2. Reactome pathways up-regulated by bevacizumab in IGROV-1 and SKOV3 models; Table S3. Reactome pathways down-regulated by bevacizumab in IGROV-1 and SKOV3 models.
