# Supplementary material for: Rewiring of Lipid Metabolism and Storage in Ovarian Cancer Cells after Anti-VEGF Therapy
Source: Cells. 2019 Dec 9;8(12):1601. doi: 10.3390/cells8121601 (PMC6953010; doi:10.3390/cells8121601)
Supplement: Supplementary file 1 [file cells-08-01601-s001.zip › Supplementary Materials 02122019/Figure S1-S6 Legends.docx]

**Figure S1.** **AMPK activation in ovarian cancer xenografts following anti-VEGF therapy** Representative pictures of pAMPK staining in experimental IGROV-1 and SKOV3 tumors (*left panel*). Semi-quantitative analysis of pAMPK expression in bevacizumab-treated tumors compared to control (*right panel*). Immunoreactivity was scored for both the intensity and the proportion of cells staining, as detailed under M&M section.

**Figure S2. Lipid content evaluation in IGROV-1 treated tumors compared to controls by NMR analysis**

Evaluation of relative amounts of lipid classes in bevacizumab-treated tumors compared to control. Dot blots show mean ± SD values (n=6 tumors/group).

**Figure S3. Lipid content evaluation in SKOV3 treated tumors compared to controls by NMR analysis**

Evaluation of relative amounts of lipid classes in bevacizumab-treated tumors compared to control. Dot blots show mean ± SD values (n=6 tumors/group).

**Figure S4. LD accumulation under hypoxia condition in ovarian cancer cells**

Representative images of IGROV-1 and SKOV3 cells cultured under normoxia or hypoxia for 48h following incubation with BODIPY 493/503 (*left panel*). LD accumulation is quantified as number of pixels for field. Columns show mean ± SD values (n=7 fields/sample), *** *P*< 0.001, t-test (*right panel*).

**Figure S5. Anti-VEGF therapy and culture under hypoxic conditions enrich for CD117^+^ tumor cells**

**A**: Flow cytometric detection of CD117^+^ CSCs in IGROV-1 tumors after anti-VEGF therapy. The histograms show the percentage of CD117^+^ cells in tumor cell cultures established from xenografts treated for 4 weeks with bevacizumab or control cultures. Data are represented as mean values ± SD of five different samples for group, ** *P*< 0.01, t-test. (*left panel*) Histogram shows the percentage of IGROV-1 and SKOV3 CD117^+^ cells following cultivation under normoxia (N) or hypoxia (H) for 72h. Data are represented as mean values ± SD of two different replicates. (*right panel*)

**B**: Quantification of LD in IGROV-1 and SKOV3 tumor cells, cultured under normoxia (N) or hypoxia (H) for 72h, by flow cytometry analysis following staining with BODIPY 493/503 dye. X-mean values are normalized to normoxia condition. Columns show mean ± SD values of two experimental replicates.

**C**: Quantification of LD in CD117^+^ cancer cells compared to total cells by flow cytometry analysis following staining with BODIPY 493/503 dye. Columns show mean ± SD values of two experimental replicates.

**Figure S6. Flow cytometric morphologic evaluation of ovarian cancer cells**

IGROV-1 and SKOV3 cell morphology following GW3965 treatment and under serum starvation condition (1% FBS) with supplementation of oleic acid (1.8 mM). Results are shown as representative dot blots of cell population.
