# Supplementary material for: Rewiring of Lipid Metabolism and Storage in Ovarian Cancer Cells after Anti-VEGF Therapy
Source: Cells. 2019 Dec 9;8(12):1601. doi: 10.3390/cells8121601 (PMC6953010; doi:10.3390/cells8121601)
Supplement: Supplementary file 1 [file cells-08-01601-s001.zip › Supplementary Materials 02122019/Table S1.docx]

**Table S1.** Summary of the lipids intra-class comparisons in Bevacizumab versus Control samples

**IGROV-1**

|  | **Chain lenght** | **Unsaturations** | **Unsaturation Index** |
| --- | --- | --- | --- |
| **Triacylglycerol** | ↓ ** | -- | ↑ * |
| **Ceramide** | -- | ↑ | ↑ |
| **Glycosyl-Ceramide** | -- | ↑ | ↑ |
| **Sphingomyelin** | ↑ | ↑ * | ↑ * |
| **Phosphatidylcholine** | -- | -- | -- |
| **Plasmanyl and Plasmenyl-PC** | ↓ * | ↓ | ↓ |
| **Phosphatidylethanolamine** | -- | -- | -- |
| **Plasmanyl and Plasmenyl-PE** | ↓ | -- | -- |
| **Diacylglycerol** | ↑ | ↑ | ↑ * |
| **Lyso-Phosphatidylcholine** | ↓ ** | ↑ | ↑ |

**SKOV3**

|  | **Chain lenght** | **Unsaturations** | **Unsaturation Index** |
| --- | --- | --- | --- |
| **Triacylglycerol** | -- | -- | -- |
| **Ceramide** | ↑ * | ↑ | ↑ |
| **Glycosyl-Ceramide** | -- | ↑ ** | ↑ *** |
| **Sphingomyelin** | ↓ | -- | -- |
| **Phosphatidylcholine** | ↓ ** | ↓ ** | ↓ ** |
| **Plasmanyl and Plasmenyl-PC** | -- | ↑ * | ↑ * |
| **Phosphatidylethanolamine** | -- | -- | -- |
| **Plasmanyl and Plasmenyl-PE** | ↓ ** | -- | -- |
| **Diacylglycerol** | -- | -- | -- |
| **Lyso-Phosphatidylcholine** | ↓ * | -- | -- |

Upward and down-ward arrows denote higher and lower values, respectively, in Bevacizumab samples respect to Control. Stars represent the level of significance (*, p < 0,05; **, p < 0,01; ***, p < 0,001), whereas missing stars denote non-significant p-values, but still lower than 0,2.
