# Supplementary material for: Rewiring of Lipid Metabolism and Storage in Ovarian Cancer Cells after Anti-VEGF Therapy
Source: Cells. 2019 Dec 9;8(12):1601. doi: 10.3390/cells8121601 (PMC6953010; doi:10.3390/cells8121601)
Supplement: Supplementary file 1 [file cells-08-01601-s001.zip › Supplementary Materials 02122019/Table S2.docx]

**Table S2.** Reactome pathways up-regulated by Bevacizumab in IGROV-1 and SKOV3 models

| **IGROV-1 model** |  |  |  |  |
| --- | --- | --- | --- | --- |
| **Pathway** | **size** | **NES** | **p-value** | **FDR q-value** |
| **REACTOME_GLYCOLYSIS** | 26 | 2.36 | 0.000 | 0.000 |
| REACTOME_GENERIC_TRANSCRIPTION_PATHWAY | 333 | 2.10 | 0.000 | 0.011 |
| REACTOME_PRE_NOTCH_TRANSCRIPTION_AND_TRANSLATION | 23 | 2.00 | 0.002 | 0.028 |
| REACTOME_SYNTHESIS_OF_PIPS_AT_THE_GOLGI_MEMBRANE | 17 | 1.94 | 0.002 | 0.043 |
| REACTOME_GLUCOSE_METABOLISM | 63 | 1.90 | 0.002 | 0.049 |
| **REACTOME_PHOSPHOLIPID_METABOLISM** | 189 | 1.89 | 0.000 | 0.047 |
| REACTOME_PRE_NOTCH_EXPRESSION_AND_PROCESSING | 38 | 1.87 | 0.000 | 0.046 |
| REACTOME_GLUCONEOGENESIS | 31 | 1.87 | 0.000 | 0.044 |
| REACTOME_TRANS_GOLGI_NETWORK_VESICLE_BUDDING | 57 | 1.80 | 0.002 | 0.075 |
| REACTOME_INSULIN_RECEPTOR_RECYCLING | 23 | 1.79 | 0.005 | 0.073 |
| REACTOME_PROTEOLYTIC_CLEAVAGE_OF_SNARE_COMPLEX_PROTEINS | 16 | 1.75 | 0.017 | 0.098 |
| REACTOME_ACYL_CHAIN_REMODELLING_OF_PS | 15 | 1.70 | 0.009 | 0.135 |
| **REACTOME_SIGNALING_BY_NOTCH** | 95 | 1.67 | 0.005 | 0.162 |
| REACTOME_GOLGI_ASSOCIATED_VESICLE_BIOGENESIS | 50 | 1.66 | 0.003 | 0.154 |
| REACTOME_GAB1_SIGNALOSOME | 36 | 1.62 | 0.017 | 0.197 |
| REACTOME_METABOLISM_OF_CARBOHYDRATES | 229 | 1.62 | 0.000 | 0.195 |
| **REACTOME_GLYCOSPHINGOLIPID_METABOLISM** | 37 | 1.61 | 0.015 | 0.190 |
| REACTOME_PI_METABOLISM | 47 | 1.60 | 0.018 | 0.190 |
| **REACTOME_GLYCEROPHOSPHOLIPID_BIOSYNTHESIS** | 79 | 1.60 | 0.008 | 0.186 |
| **REACTOME_TRIGLYCERIDE_BIOSYNTHESIS** | 36 | 1.57 | 0.015 | 0.216 |
| **REACTOME_METABOLISM_OF_LIPIDS_AND_LIPOPROTEINS** | 461 | 1.56 | 0.000 | 0.211 |
|  |  |  |  |  |
| **SKOV3 model** |  |  |  |  |
| **Pathway** | **size** | **NES** | **p-value** | **FDR q-value** |
| **REACTOME_CHONDROITIN_SULFATE_BIOSYNTHESIS** | 19 | 2.20 | 0.000 | 0.007 |
| **REACTOME_CHONDROITIN_SULFATE_DERMATAN_SULFATE_METABOLISM** | 47 | 2.14 | 0.000 | 0.008 |
| REACTOME_OLFACTORY_SIGNALING_PATHWAY | 305 | 2.08 | 0.000 | 0.014 |
| REACTOME_CLASS_B_2_SECRETIN_FAMILY_RECEPTORS | 82 | 1.88 | 0.000 | 0.100 |
| REACTOME_NCAM1_INTERACTIONS | 39 | 1.86 | 0.002 | 0.095 |
| **REACTOME_GLYCOSAMINOGLYCAN_METABOLISM** | 106 | 1.84 | 0.000 | 0.093 |
| REACTOME_PHOSPHORYLATION_OF_CD3_AND_TCR_ZETA_CHAINS | 16 | 1.79 | 0.005 | 0.135 |
| REACTOME_A_TETRASACCHARIDE_LINKER_SEQUENCE_IS_REQUIRED_FOR_GAG_SYNTHESIS | 25 | 1.78 | 0.009 | 0.128 |
| REACTOME_COMPLEMENT_CASCADE | 29 | 1.77 | 0.002 | 0.125 |
| REACTOME_SYNTHESIS_SECRETION_AND_DEACYLATION_OF_GHRELIN | 15 | 1.73 | 0.009 | 0.149 |
| REACTOME_NCAM_SIGNALING_FOR_NEURITE_OUT_GROWTH | 64 | 1.73 | 0.002 | 0.138 |
| REACTOME_CD28_DEPENDENT_PI3K_AKT_SIGNALING | 21 | 1.72 | 0.013 | 0.140 |
| **REACTOME_SIGNALING_BY_NOTCH** | 95 | 1.71 | 0.002 | 0.141 |
| REACTOME_NUCLEAR_SIGNALING_BY_ERBB4 | 38 | 1.71 | 0.009 | 0.136 |
| REACTOME_SIGNALING_BY_PDGF | 117 | 1.68 | 0.002 | 0.149 |
| **REACTOME_SIGNALING_BY_NOTCH1** | 67 | 1.65 | 0.009 | 0.168 |
| REACTOME_G_ALPHA_S_SIGNALLING_EVENTS | 119 | 1.64 | 0.000 | 0.171 |
| REACTOME_GPCR_LIGAND_BINDING | 381 | 1.63 | 0.000 | 0.177 |
| REACTOME_KERATAN_SULFATE_BIOSYNTHESIS | 25 | 1.61 | 0.018 | 0.193 |
| REACTOME_CIRCADIAN_CLOCK | 50 | 1.60 | 0.012 | 0.202 |
| REACTOME_NEURONAL_SYSTEM | 275 | 1.59 | 0.000 | 0.206 |
| REACTOME_TGF_BETA_RECEPTOR_SIGNALING_ACTIVATES_SMADS | 24 | 1.59 | 0.016 | 0.199 |
| REACTOME_HEPARAN_SULFATE_HEPARIN_HS_GAG_METABOLISM | 50 | 1.58 | 0.009 | 0.199 |
| REACTOME_NOTCH1_INTRACELLULAR_DOMAIN_REGULATES_TRANSCRIPTION | 44 | 1.58 | 0.019 | 0.192 |
| REACTOME_ION_CHANNEL_TRANSPORT | 54 | 1.55 | 0.016 | 0.210 |
| REACTOME_POTASSIUM_CHANNELS | 97 | 1.54 | 0.004 | 0.205 |
| REACTOME_PHASE1_FUNCTIONALIZATION_OF_COMPOUNDS | 68 | 1.54 | 0.014 | 0.204 |
| REACTOME_EXTRACELLULAR_MATRIX_ORGANIZATION | 81 | 1.50 | 0.009 | 0.214 |
| REACTOME_TRANSMISSION_ACROSS_CHEMICAL_SYNAPSES | 183 | 1.48 | 0.009 | 0.230 |
| REACTOME_DEVELOPMENTAL_BIOLOGY | 380 | 1.48 | 0.000 | 0.226 |

Results of gene set enrichment analysis. Size: number of genes in the expression dataset belonging to the pathway; NES: normalized enrichment score; FDR: false discovery rate. Pathways directly cited in the results section are elicited in bold.
