# Supplementary material for: Rewiring of Lipid Metabolism and Storage in Ovarian Cancer Cells after Anti-VEGF Therapy
Source: Cells. 2019 Dec 9;8(12):1601. doi: 10.3390/cells8121601 (PMC6953010; doi:10.3390/cells8121601)
Supplement: Supplementary file 1 [file cells-08-01601-s001.zip › Supplementary Materials 02122019/Table S3.docx]

**Table S3.** Reactome pathways down-regulated by Bevacizumab in IGROV-1 and SKOV3 models

| **IGROV-1 model** |  |  |  | |  | |  |  |
| --- | --- | --- | --- | --- | --- | --- | --- | --- |
| **Pathway** | **size** | **NES** | | **p-value** | | **FDR q-value** | | |
| REACTOME_MITOTIC_M_M_G1_PHASES | 161 | -2.86 | | 0.000 | | 0.000 | | |
| **REACTOME_DNA_REPLICATION** | 181 | -2.84 | | 0.000 | | 0.000 | | |
| REACTOME_MITOTIC_PROMETAPHASE | 79 | -2.66 | | 0.000 | | 0.000 | | |
| REACTOME_M_G1_TRANSITION | 78 | -2.60 | | 0.000 | | 0.000 | | |
| REACTOME_DEPOSITION_OF_NEW_CENPA_CONTAINING_NUCLEOSOMES.. (REACT_22186) | 60 | -2.58 | | 0.000 | | 0.000 | | |
| REACTOME_MEIOTIC_RECOMBINATION | 80 | -2.50 | | 0.000 | | 0.000 | | |
| REACTOME_CELL_CYCLE_CHECKPOINTS | 111 | -2.49 | | 0.000 | | 0.000 | | |
| REACTOME_G1_S_TRANSITION | 106 | -2.48 | | 0.000 | | 0.000 | | |
| **REACTOME_SYNTHESIS_OF_DNA** | 90 | -2.48 | | 0.000 | | 0.000 | | |
| REACTOME_CELL_CYCLE | 391 | -2.44 | | 0.000 | | 0.000 | | |
| REACTOME_CELL_CYCLE_MITOTIC | 300 | -2.43 | | 0.000 | | 0.000 | | |
| REACTOME_REGULATION_OF_MITOTIC_CELL_CYCLE | 77 | -2.38 | | 0.000 | | 0.000 | | |
| REACTOME_S_PHASE | 106 | -2.38 | | 0.000 | | 0.000 | | |
| REACTOME_ASSEMBLY_OF_THE_PRE_REPLICATIVE_COMPLEX | 63 | -2.38 | | 0.000 | | 0.000 | | |
| REACTOME_APC_C_CDC20_MEDIATED_DEGRADATION_OF_MITOTIC_PROTEINS | 65 | -2.37 | | 0.000 | | 0.000 | | |
| REACTOME_ACTIVATION_OF_THE_PRE_REPLICATIVE_COMPLEX | 30 | -2.32 | | 0.000 | | 0.000 | | |
| REACTOME_RNA_POL_I_PROMOTER_OPENING | 58 | -2.32 | | 0.000 | | 0.000 | | |
| REACTOME_MITOTIC_G1_G1_S_PHASES | 130 | -2.30 | | 0.000 | | 0.000 | | |
| REACTOME_CDT1_ASSOCIATION_WITH_THE_CDC6_ORC_ORIGIN_COMPLEX | 54 | -2.29 | | 0.000 | | 0.000 | | |
| REACTOME_VIF_MEDIATED_DEGRADATION_OF_APOBEC3G | 47 | -2.26 | | 0.000 | | 0.000 | | |
| REACTOME_ORC1_REMOVAL_FROM_CHROMATIN | 65 | -2.25 | | 0.000 | | 0.000 | | |
| REACTOME_CHROMOSOME_MAINTENANCE | 116 | -2.24 | | 0.000 | | 0.001 | | |
| REACTOME_CDK_MEDIATED_PHOSPHORYLATION_AND_REMOVAL_OF_CDC6 | 46 | -2.23 | | 0.000 | | 0.000 | | |
| REACTOME_SCF_BETA_TRCP_MEDIATED_DEGRADATION_OF_EMI1 | 49 | -2.21 | | 0.000 | | 0.001 | | |
| REACTOME_AMYLOIDS | 78 | -2.20 | | 0.000 | | 0.001 | | |
| **REACTOME_DNA_STRAND_ELONGATION** | 30 | -2.19 | | 0.000 | | 0.001 | | |
| REACTOME_ACTIVATED_POINT_MUTANTS_OF_FGFR2 | 16 | -2.18 | | 0.000 | | 0.001 | | |
| REACTOME_MEIOSIS | 108 | -2.17 | | 0.000 | | 0.001 | | |
| REACTOME_AUTODEGRADATION_OF_CDH1_BY_CDH1_APC_C | 56 | -2.17 | | 0.000 | | 0.001 | | |
| REACTOME_ACTIVATION_OF_NF_KAPPAB_IN_B_CELLS | 61 | -2.16 | | 0.000 | | 0.001 | | |
| REACTOME_APC_C_CDH1_MEDIATED_DEGRADATION_OF_CDC20_AND.. (REACT_6761) | 64 | -2.15 | | 0.000 | | 0.001 | | |
| REACTOME_G2_M_CHECKPOINTS | 41 | -2.14 | | 0.000 | | 0.001 | | |
| REACTOME_ACTIVATION_OF_ATR_IN_RESPONSE_TO_REPLICATION_STRESS | 35 | -2.13 | | 0.000 | | 0.001 | | |
| REACTOME_CROSS_PRESENTATION_OF_SOLUBLE_EXOGENOUS_ANTIGENS_ENDOSOMES | 47 | -2.11 | | 0.003 | | 0.002 | | |
| REACTOME_P53_INDEPENDENT_G1_S_DNA_DAMAGE_CHECKPOINT | 48 | -2.10 | | 0.000 | | 0.002 | | |
| REACTOME_DESTABILIZATION_OF_MRNA_BY_AUF1_HNRNP_D0 | 50 | -2.09 | | 0.000 | | 0.002 | | |
| REACTOME_SCFSKP2_MEDIATED_DEGRADATION_OF_P27_P21 | 53 | -2.08 | | 0.000 | | 0.002 | | |
| REACTOME_AUTODEGRADATION_OF_THE_E3_UBIQUITIN_LIGASE_COP1 | 47 | -2.07 | | 0.000 | | 0.002 | | |
| REACTOME_TELOMERE_MAINTENANCE | 75 | -2.07 | | 0.000 | | 0.002 | | |
| **REACTOME_REGULATION_OF_APOPTOSIS** | 56 | -2.06 | | 0.000 | | 0.002 | | |
| REACTOME_SIGNALING_BY_WNT | 62 | -2.04 | | 0.000 | | 0.002 | | |
| REACTOME_P53_DEPENDENT_G1_DNA_DAMAGE_RESPONSE | 53 | -2.04 | | 0.000 | | 0.002 | | |
| REACTOME_RNA_POL_I_TRANSCRIPTION | 81 | -2.03 | | 0.000 | | 0.003 | | |
| REACTOME_CYCLIN_E_ASSOCIATED_EVENTS_DURING_G1_S_TRANSITION | 62 | -1.98 | | 0.000 | | 0.005 | | |
| REACTOME_E2F_MEDIATED_REGULATION_OF_DNA_REPLICATION | 33 | -1.96 | | 0.000 | | 0.005 | | |
| REACTOME_DOUBLE_STRAND_BREAK_REPAIR | 21 | -1.95 | | 0.000 | | 0.006 | | |
| REACTOME_PACKAGING_OF_TELOMERE_ENDS | 48 | -1.93 | | 0.000 | | 0.007 | | |
| REACTOME_REGULATION_OF_ORNITHINE_DECARBOXYLASE_ODC | 48 | -1.93 | | 0.000 | | 0.008 | | |
| REACTOME_APC_CDC20_MEDIATED_DEGRADATION_OF_NEK2A | 21 | -1.92 | | 0.004 | | 0.008 | | |
| REACTOME_ANTIGEN_PROCESSING_CROSS_PRESENTATION | 72 | -1.90 | | 0.000 | | 0.009 | | |
| REACTOME_MEIOTIC_SYNAPSIS | 71 | -1.89 | | 0.000 | | 0.010 | | |
| REACTOME_FANCONI_ANEMIA_PATHWAY | 19 | -1.88 | | 0.005 | | 0.011 | | |
| **REACTOME_APOPTOSIS** | 142 | -1.88 | | 0.000 | | 0.011 | | |
| REACTOME_HOMOLOGOUS_RECOMBINATION_REPAIR_OF_REPLICATION.. (REACT_1587) | 15 | -1.85 | | 0.009 | | 0.015 | | |
| REACTOME_METABOLISM_OF_NON_CODING_RNA | 47 | -1.84 | | 0.000 | | 0.015 | | |
| REACTOME_TRANSPORT_TO_THE_GOLGI_AND_SUBSEQUENT_MODIFICATION | 33 | -1.83 | | 0.002 | | 0.015 | | |
| REACTOME_SHC_MEDIATED_CASCADE | 28 | -1.83 | | 0.009 | | 0.016 | | |
| REACTOME_FGFR_LIGAND_BINDING_AND_ACTIVATION | 22 | -1.82 | | 0.005 | | 0.017 | | |
| REACTOME_G0_AND_EARLY_G1 | 23 | -1.81 | | 0.002 | | 0.018 | | |
| REACTOME_CYCLIN_A_B1_ASSOCIATED_EVENTS_DURING_G2_M_TRANSITION | 15 | -1.79 | | 0.009 | | 0.022 | | |
| REACTOME_ER_PHAGOSOME_PATHWAY | 58 | -1.77 | | 0.002 | | 0.024 | | |
| REACTOME_G1_S_SPECIFIC_TRANSCRIPTION | 17 | -1.75 | | 0.004 | | 0.028 | | |
| REACTOME_INHIBITION_OF_THE_PROTEOLYTIC_ACTIVITY_OF_APC_C.. (REACT_1041) | 18 | -1.75 | | 0.007 | | 0.028 | | |
| REACTOME_ANTIVIRAL_MECHANISM_BY_IFN_STIMULATED_GENES | 63 | -1.73 | | 0.000 | | 0.031 | | |
| REACTOME_HOST_INTERACTIONS_OF_HIV_FACTORS | 117 | -1.73 | | 0.000 | | 0.031 | | |
| REACTOME_APC_C_CDC20_MEDIATED_DEGRADATION_OF_CYCLIN_B | 19 | -1.72 | | 0.009 | | 0.034 | | |
| **REACTOME_APOPTOTIC_EXECUTION_PHASE** | 51 | -1.71 | | 0.000 | | 0.035 | | |
| REACTOME_DOWNSTREAM_SIGNALING_EVENTS_OF_B_CELL_RECEPTOR_BCR | 92 | -1.71 | | 0.000 | | 0.034 | | |
| REACTOME_FRS2_MEDIATED_CASCADE | 36 | -1.69 | | 0.014 | | 0.042 | | |
| REACTOME_LAGGING_STRAND_SYNTHESIS | 19 | -1.67 | | 0.017 | | 0.047 | | |
| REACTOME_ACTIVATION_OF_KAINATE_RECEPTORS_UPON_GLUTAMATE_BINDING | 30 | -1.66 | | 0.019 | | 0.050 | | |
| REACTOME_G_PROTEIN_ACTIVATION | 26 | -1.64 | | 0.019 | | 0.057 | | |
| REACTOME_RNA_POL_I_RNA_POL_III_AND_MITOCHONDRIAL_TRANSCRIPTION | 113 | -1.63 | | 0.000 | | 0.059 | | |
| REACTOME_EXTENSION_OF_TELOMERES | 27 | -1.63 | | 0.016 | | 0.061 | | |
| REACTOME_REGULATION_OF_MRNA_STABILITY_BY_PROTEINS_THAT_BIND.. (REACT_24994) | 81 | -1.60 | | 0.008 | | 0.071 | | |
| REACTOME_G_ALPHA_I_SIGNALLING_EVENTS | 186 | -1.59 | | 0.000 | | 0.076 | | |
| REACTOME_PREFOLDIN_MEDIATED_TRANSFER_OF_SUBSTRATE_TO_CCT_TRIC | 27 | -1.59 | | 0.019 | | 0.076 | | |
| REACTOME_SIGNALING_BY_THE_B_CELL_RECEPTOR_BCR | 121 | -1.58 | | 0.000 | | 0.077 | | |
| REACTOME_METABOLISM_OF_RNA | 252 | -1.56 | | 0.000 | | 0.088 | | |
| REACTOME_HIV_INFECTION | 184 | -1.54 | | 0.000 | | 0.095 | | |
| REACTOME_TRANSCRIPTION | 191 | -1.51 | | 0.000 | | 0.108 | | |
| REACTOME_PEPTIDE_LIGAND_BINDING_RECEPTORS | 174 | -1.49 | | 0.000 | | 0.116 | | |
| **REACTOME_RESPIRATORY_ELECTRON_TRANSPORT_ATP_SYNTHESIS_BY.. (REACT_6305)** | 84 | -1.48 | | 0.007 | | 0.120 | | |
| **REACTOME_DNA_REPAIR** | 101 | -1.47 | | 0.013 | | 0.128 | | |
| REACTOME_CLASS_I_MHC_MEDIATED_ANTIGEN_PROCESSING_PRESENTATION | 231 | -1.43 | | 0.000 | | 0.150 | | |
| REACTOME_CLASS_A1_RHODOPSIN_LIKE_RECEPTORS | 285 | -1.41 | | 0.003 | | 0.161 | | |
| REACTOME_GPCR_LIGAND_BINDING | 381 | -1.35 | | 0.004 | | 0.208 | | |
|  |  |  | |  | |  | | |
| **SKOV3 model** |  |  | |  | |  | | |
| **pathway** | **size** | **NES** | | **p-value** | | **FDR q-value** | | |
| **REACTOME_DNA_REPLICATION** | 181 | -3.60 | | 0.000 | | 0.000 | | |
| REACTOME_MITOTIC_M_M_G1_PHASES | 161 | -3.48 | | 0.000 | | 0.000 | | |
| REACTOME_G1_S_TRANSITION | 106 | -3.40 | | 0.000 | | 0.000 | | |
| **REACTOME_RESPIRATORY_ELECTRON_TRANSPORT_ATP_SYNTHESIS_BY.. (REACT_6305)** | 84 | -3.37 | | 0.000 | | 0.000 | | |
| **REACTOME_SYNTHESIS_OF_DNA** | 90 | -3.35 | | 0.000 | | 0.000 | | |
| REACTOME_S_PHASE | 106 | -3.34 | | 0.000 | | 0.000 | | |
| REACTOME_TCA_CYCLE_AND_RESPIRATORY_ELECTRON_TRANSPORT | 120 | -3.34 | | 0.000 | | 0.000 | | |
| REACTOME_MITOTIC_G1_G1_S_PHASES | 130 | -3.31 | | 0.000 | | 0.000 | | |
| REACTOME_M_G1_TRANSITION | 78 | -3.28 | | 0.000 | | 0.000 | | |
| REACTOME_CELL_CYCLE_CHECKPOINTS | 111 | -3.22 | | 0.000 | | 0.000 | | |
| REACTOME_RESPIRATORY_ELECTRON_TRANSPORT | 67 | -3.19 | | 0.000 | | 0.000 | | |
| REACTOME_CELL_CYCLE_MITOTIC | 300 | -3.18 | | 0.000 | | 0.000 | | |
| REACTOME_ASSEMBLY_OF_THE_PRE_REPLICATIVE_COMPLEX | 63 | -3.15 | | 0.000 | | 0.000 | | |
| REACTOME_ORC1_REMOVAL_FROM_CHROMATIN | 65 | -3.10 | | 0.000 | | 0.000 | | |
| REACTOME_CELL_CYCLE | 391 | -3.07 | | 0.000 | | 0.000 | | |
| REACTOME_G2_M_CHECKPOINTS | 41 | -2.97 | | 0.000 | | 0.000 | | |
| REACTOME_REGULATION_OF_ORNITHINE_DECARBOXYLASE_ODC | 48 | -2.93 | | 0.000 | | 0.000 | | |
| REACTOME_REGULATION_OF_MITOTIC_CELL_CYCLE | 77 | -2.93 | | 0.000 | | 0.000 | | |
| REACTOME_E2F_MEDIATED_REGULATION_OF_DNA_REPLICATION | 33 | -2.92 | | 0.000 | | 0.000 | | |
| REACTOME_CDT1_ASSOCIATION_WITH_THE_CDC6_ORC_ORIGIN_COMPLEX | 54 | -2.89 | | 0.000 | | 0.000 | | |
| REACTOME_ACTIVATION_OF_ATR_IN_RESPONSE_TO_REPLICATION_STRESS | 35 | -2.86 | | 0.000 | | 0.000 | | |
| **REACTOME_DNA_STRAND_ELONGATION** | 30 | -2.85 | | 0.000 | | 0.000 | | |
| REACTOME_APC_C_CDH1_MEDIATED_DEGRADATION_OF_CDC20_AND.. (REACT_6761) | 64 | -2.85 | | 0.000 | | 0.000 | | |
| REACTOME_APC_C_CDC20_MEDIATED_DEGRADATION_OF_MITOTIC_PROTEINS | 65 | -2.85 | | 0.000 | | 0.000 | | |
| REACTOME_SCFSKP2_MEDIATED_DEGRADATION_OF_P27_P21 | 53 | -2.84 | | 0.000 | | 0.000 | | |
| REACTOME_CYCLIN_E_ASSOCIATED_EVENTS_DURING_G1_S_TRANSITION_ | 62 | -2.84 | | 0.000 | | 0.000 | | |
| REACTOME_CDK_MEDIATED_PHOSPHORYLATION_AND_REMOVAL_OF_CDC6 | 46 | -2.82 | | 0.000 | | 0.000 | | |
| REACTOME_MITOTIC_PROMETAPHASE | 79 | -2.77 | | 0.000 | | 0.000 | | |
| REACTOME_P53_INDEPENDENT_G1_S_DNA_DAMAGE_CHECKPOINT | 48 | -2.76 | | 0.000 | | 0.000 | | |
| REACTOME_HIV_INFECTION | 184 | -2.76 | | 0.000 | | 0.000 | | |
| REACTOME_MITOCHONDRIAL_PROTEIN_IMPORT | 48 | -2.75 | | 0.000 | | 0.000 | | |
| REACTOME_AUTODEGRADATION_OF_CDH1_BY_CDH1_APC_C | 56 | -2.75 | | 0.000 | | 0.000 | | |
| REACTOME_HOST_INTERACTIONS_OF_HIV_FACTORS | 117 | -2.75 | | 0.000 | | 0.000 | | |
| REACTOME_DESTABILIZATION_OF_MRNA_BY_AUF1_HNRNP_D0 | 50 | -2.73 | | 0.000 | | 0.000 | | |
| REACTOME_ACTIVATION_OF_THE_PRE_REPLICATIVE_COMPLEX | 30 | -2.73 | | 0.000 | | 0.000 | | |
| REACTOME_G1_S_SPECIFIC_TRANSCRIPTION | 17 | -2.71 | | 0.000 | | 0.000 | | |
| REACTOME_VIF_MEDIATED_DEGRADATION_OF_APOBEC3G | 47 | -2.66 | | 0.000 | | 0.000 | | |
| REACTOME_SCF_BETA_TRCP_MEDIATED_DEGRADATION_OF_EMI1 | 49 | -2.63 | | 0.000 | | 0.000 | | |
| REACTOME_CROSS_PRESENTATION_OF_SOLUBLE_EXOGENOUS_ANTIGENS_ENDOSOMES | 47 | -2.62 | | 0.000 | | 0.000 | | |
| REACTOME_P53_DEPENDENT_G1_DNA_DAMAGE_RESPONSE | 53 | -2.60 | | 0.000 | | 0.000 | | |
| REACTOME_AUTODEGRADATION_OF_THE_E3_UBIQUITIN_LIGASE_COP1 | 47 | -2.58 | | 0.000 | | 0.000 | | |
| REACTOME_MRNA_SPLICING_MINOR_PATHWAY | 40 | -2.57 | | 0.000 | | 0.000 | | |
| REACTOME_ER_PHAGOSOME_PATHWAY | 58 | -2.56 | | 0.000 | | 0.000 | | |
| REACTOME_METABOLISM_OF_AMINO_ACIDS_AND_DERIVATIVES | 189 | -2.51 | | 0.000 | | 0.000 | | |
| REACTOME_REGULATION_OF_MRNA_STABILITY_BY_PROTEINS_THAT_BIND.. (REACT_24994) | 81 | -2.50 | | 0.000 | | 0.000 | | |
| **REACTOME_REGULATION_OF_APOPTOSIS** | 56 | -2.49 | | 0.000 | | 0.000 | | |
| REACTOME_EXTENSION_OF_TELOMERES | 27 | -2.48 | | 0.000 | | 0.000 | | |
| REACTOME_PROCESSING_OF_CAPPED_INTRON_CONTAINING_PRE_MRNA | 132 | -2.45 | | 0.000 | | 0.000 | | |
| REACTOME_MRNA_SPLICING | 104 | -2.45 | | 0.000 | | 0.000 | | |
| REACTOME_G0_AND_EARLY_G1 | 23 | -2.43 | | 0.000 | | 0.000 | | |
| REACTOME_MRNA_PROCESSING | 151 | -2.43 | | 0.000 | | 0.000 | | |
| REACTOME_ACTIVATION_OF_NF_KAPPAB_IN_B_CELLS | 61 | -2.40 | | 0.000 | | 0.000 | | |
| REACTOME_LAGGING_STRAND_SYNTHESIS | 19 | -2.39 | | 0.000 | | 0.000 | | |
| REACTOME_CITRIC_ACID_CYCLE_TCA_CYCLE | 19 | -2.39 | | 0.000 | | 0.000 | | |
| REACTOME_SIGNALING_BY_WNT | 62 | -2.38 | | 0.000 | | 0.000 | | |
| REACTOME_ANTIGEN_PROCESSING_CROSS_PRESENTATION | 72 | -2.37 | | 0.000 | | 0.000 | | |
| REACTOME_METABOLISM_OF_NON_CODING_RNA | 47 | -2.37 | | 0.000 | | 0.000 | | |
| **REACTOME_DNA_REPAIR** | 101 | -2.32 | | 0.000 | | 0.000 | | |
| REACTOME_METABOLISM_OF_RNA | 252 | -2.28 | | 0.000 | | 0.000 | | |
| REACTOME_CYCLIN_A_B1_ASSOCIATED_EVENTS_DURING_G2_M_TRANSITION | 15 | -2.28 | | 0.000 | | 0.000 | | |
| REACTOME_CHROMOSOME_MAINTENANCE | 116 | -2.23 | | 0.000 | | 0.000 | | |
| REACTOME_HIV_LIFE_CYCLE | 105 | -2.22 | | 0.000 | | 0.000 | | |
| REACTOME_RNA_POL_III_TRANSCRIPTION_TERMINATION | 19 | -2.20 | | 0.000 | | 0.000 | | |
| REACTOME_RNA_POL_III_CHAIN_ELONGATION | 17 | -2.18 | | 0.000 | | 0.000 | | |
| REACTOME_KINESINS | 24 | -2.18 | | 0.000 | | 0.000 | | |
| REACTOME_PYRUVATE_METABOLISM_AND_CITRIC_ACID_TCA_CYCLE | 40 | -2.17 | | 0.000 | | 0.000 | | |
| REACTOME_METABOLISM_OF_VITAMINS_AND_COFACTORS | 50 | -2.14 | | 0.000 | | 0.000 | | |
| REACTOME_BRANCHED_CHAIN_AMINO_ACID_CATABOLISM | 17 | -2.13 | | 0.000 | | 0.001 | | |
| REACTOME_METABOLISM_OF_NUCLEOTIDES | 69 | -2.13 | | 0.000 | | 0.000 | | |
| REACTOME_LATE_PHASE_OF_HIV_LIFE_CYCLE | 92 | -2.12 | | 0.000 | | 0.001 | | |
| REACTOME_APC_CDC20_MEDIATED_DEGRADATION_OF_NEK2A | 21 | -2.12 | | 0.000 | | 0.001 | | |
| REACTOME_TELOMERE_MAINTENANCE | 75 | -2.11 | | 0.000 | | 0.001 | | |
| REACTOME_RNA_POL_II_PRE_TRANSCRIPTION_EVENTS | 51 | -2.10 | | 0.000 | | 0.001 | | |
| REACTOME_PROCESSIVE_SYNTHESIS_ON_THE_LAGGING_STRAND | 15 | -2.10 | | 0.002 | | 0.001 | | |
| REACTOME_PROCESSING_OF_CAPPED_INTRONLESS_PRE_MRNA | 23 | -2.09 | | 0.000 | | 0.001 | | |
| REACTOME_DOUBLE_STRAND_BREAK_REPAIR | 21 | -2.06 | | 0.000 | | 0.001 | | |
| REACTOME_METABOLISM_OF_PROTEINS | 418 | -2.06 | | 0.000 | | 0.001 | | |
| REACTOME_APC_C_CDC20_MEDIATED_DEGRADATION_OF_CYCLIN_B | 19 | -2.06 | | 0.000 | | 0.001 | | |
| REACTOME_TRANSCRIPTION_COUPLED_NER_TC_NER | 43 | -2.05 | | 0.000 | | 0.001 | | |
| **REACTOME_APOPTOSIS** | 142 | -2.04 | | 0.000 | | 0.001 | | |
| REACTOME_RNA_POL_II_TRANSCRIPTION_PRE_INITIATION_AND_PROMOTER_OPENING | 39 | -2.03 | | 0.000 | | 0.001 | | |
| REACTOME_RNA_POL_III_TRANSCRIPTION_INITIATION_FROM_TYPE_3_PROMOTER | 26 | -2.03 | | 0.000 | | 0.001 | | |
| REACTOME_BIOSYNTHESIS_OF_THE_N_GLYCAN_PRECURSOR_DOLICHOL.. (REACT_22433) | 28 | -2.02 | | 0.000 | | 0.001 | | |
| REACTOME_TRNA_AMINOACYLATION | 42 | -2.02 | | 0.000 | | 0.001 | | |
| REACTOME_TRANSCRIPTION | 191 | -2.02 | | 0.000 | | 0.001 | | |
| REACTOME_METABOLISM_OF_MRNA | 208 | -2.01 | | 0.000 | | 0.002 | | |
| REACTOME_RNA_POL_II_TRANSCRIPTION | 93 | -2.00 | | 0.000 | | 0.002 | | |
| REACTOME_HOMOLOGOUS_RECOMBINATION_REPAIR_OF_REPLICATION.. (REACT_1587) | 15 | -2.00 | | 0.002 | | 0.002 | | |
| REACTOME_INHIBITION_OF_THE_PROTEOLYTIC_ACTIVITY_OF_APC_C.. (REACT_1041) | 18 | -1.99 | | 0.002 | | 0.002 | | |
| REACTOME_ANTIVIRAL_MECHANISM_BY_IFN_STIMULATED_GENES | 63 | -1.99 | | 0.000 | | 0.002 | | |
| REACTOME_RNA_POL_III_TRANSCRIPTION | 33 | -1.99 | | 0.000 | | 0.002 | | |
| REACTOME_ABORTIVE_ELONGATION_OF_HIV1_TRANSCRIPT_IN_THE_ABSENCE_OF_TAT | 19 | -1.98 | | 0.002 | | 0.002 | | |
| REACTOME_ASPARAGINE_N_LINKED_GLYCOSYLATION | 80 | -1.97 | | 0.000 | | 0.002 | | |
| REACTOME_DOWNSTREAM_SIGNALING_EVENTS_OF_B_CELL_RECEPTOR_BCR | 92 | -1.96 | | 0.000 | | 0.002 | | |
| REACTOME_FANCONI_ANEMIA_PATHWAY | 19 | -1.96 | | 0.000 | | 0.002 | | |
| REACTOME_ANTIGEN_PROCESSING_UBIQUITINATION_PROTEASOME_DEGRADATION | 194 | -1.95 | | 0.000 | | 0.003 | | |
| REACTOME_RNA_POL_III_TRANSCRIPTION_INITIATION_FROM_TYPE_2_PROMOTER | 23 | -1.91 | | 0.000 | | 0.004 | | |
| REACTOME_PHOSPHORYLATION_OF_THE_APC_C | 17 | -1.91 | | 0.004 | | 0.004 | | |
| REACTOME_CLASS_I_MHC_MEDIATED_ANTIGEN_PROCESSING_PRESENTATION | 231 | -1.89 | | 0.000 | | 0.004 | | |
| REACTOME_DEPOSITION_OF_NEW_CENPA_CONTAINING_NUCLEOSOMES.. (REACT_22186) | 60 | -1.86 | | 0.000 | | 0.006 | | |
| REACTOME_MHC_CLASS_II_ANTIGEN_PRESENTATION | 89 | -1.84 | | 0.000 | | 0.007 | | |
| REACTOME_LYSOSOME_VESICLE_BIOGENESIS | 23 | -1.83 | | 0.007 | | 0.007 | | |
| REACTOME_MICRORNA_MIRNA_BIOGENESIS | 18 | -1.83 | | 0.004 | | 0.008 | | |
| REACTOME_MITOCHONDRIAL_TRNA_AMINOACYLATION | 21 | -1.82 | | 0.004 | | 0.008 | | |
| REACTOME_NUCLEOTIDE_EXCISION_REPAIR | 48 | -1.81 | | 0.002 | | 0.008 | | |
| REACTOME_GLUTATHIONE_CONJUGATION | 22 | -1.81 | | 0.007 | | 0.008 | | |
| REACTOME_FORMATION_OF_RNA_POL_II_ELONGATION_COMPLEX_ | 35 | -1.78 | | 0.002 | | 0.011 | | |
| REACTOME_DEADENYLATION_DEPENDENT_MRNA_DECAY | 42 | -1.78 | | 0.004 | | 0.011 | | |
| REACTOME_SIGNALING_BY_THE_B_CELL_RECEPTOR_BCR | 121 | -1.77 | | 0.000 | | 0.011 | | |
| REACTOME_FORMATION_OF_THE_HIV1_EARLY_ELONGATION_COMPLEX | 28 | -1.77 | | 0.005 | | 0.011 | | |
| REACTOME_ELONGATION_ARREST_AND_RECOVERY | 24 | -1.77 | | 0.011 | | 0.011 | | |
| REACTOME_SYNTHESIS_AND_INTERCONVERSION_OF_NUCLEOTIDE_DI_AND_TRIPHOSPHATES | 17 | -1.77 | | 0.004 | | 0.011 | | |
| REACTOME_RNA_POL_I_RNA_POL_III_AND_MITOCHONDRIAL_TRANSCRIPTION | 113 | -1.77 | | 0.000 | | 0.011 | | |
| REACTOME_MRNA_CAPPING | 28 | -1.76 | | 0.005 | | 0.012 | | |
| REACTOME_ASSOCIATION_OF_TRIC_CCT_WITH_TARGET_PROTEINS_DURING_BIOSYNTHESIS | 26 | -1.75 | | 0.009 | | 0.013 | | |
| REACTOME_BASE_EXCISION_REPAIR | 19 | -1.72 | | 0.018 | | 0.016 | | |
| REACTOME_FORMATION_OF_THE_TERNARY_COMPLEX_AND_SUBSEQUENTLY.. (REACT_1079) | 48 | -1.71 | | 0.000 | | 0.018 | | |
| REACTOME_CONVERSION_FROM_APC_C_CDC20_TO_APC_C_CDH1_IN_LATE_ANAPHASE | 16 | -1.70 | | 0.020 | | 0.018 | | |
| REACTOME_RESOLUTION_OF_AP_SITES_VIA_THE_MULTIPLE_NUCLEOTIDE.. ( REACT_1128) | 17 | -1.70 | | 0.004 | | 0.018 | | |
| REACTOME_THE_ROLE_OF_NEF_IN_HIV1_REPLICATION_AND_DISEASE_PATHOGENESIS | 27 | -1.69 | | 0.013 | | 0.020 | | |
| REACTOME_CYTOSOLIC_TRNA_AMINOACYLATION | 24 | -1.69 | | 0.018 | | 0.020 | | |
| REACTOME_NEF_MEDIATES_DOWN_MODULATION_OF_CELL_SURFACE.. (REACT_11149) | 20 | -1.66 | | 0.013 | | 0.024 | | |
| REACTOME_PURINE_METABOLISM | 33 | -1.66 | | 0.013 | | 0.023 | | |
| REACTOME_RNA_POL_I_TRANSCRIPTION_INITIATION | 23 | -1.66 | | 0.013 | | 0.024 | | |
| REACTOME_POST_TRANSLATIONAL_PROTEIN_MODIFICATION | 178 | -1.66 | | 0.000 | | 0.023 | | |
| REACTOME_NEP_NS2_INTERACTS_WITH_THE_CELLULAR_EXPORT_MACHINERY | 26 | -1.66 | | 0.019 | | 0.024 | | |
| REACTOME_TRANSPORT_OF_MATURE_MRNA_DERIVED_FROM_AN_INTRONLESS_TRANSCRIPT | 31 | -1.65 | | 0.013 | | 0.026 | | |
| REACTOME_G1_PHASE | 35 | -1.64 | | 0.018 | | 0.027 | | |
| REACTOME_FORMATION_OF_TRANSCRIPTION_COUPLED_NER_TC_NER_REPAIR_COMPLEX | 28 | -1.63 | | 0.018 | | 0.028 | | |
| REACTOME_INTERACTIONS_OF_VPR_WITH_HOST_CELLULAR_PROTEINS | 31 | -1.63 | | 0.014 | | 0.029 | | |
| REACTOME_ACTIVATION_OF_THE_MRNA_UPON_BINDING_OF_THE_CAP.. (REACT_1258) | 56 | -1.62 | | 0.011 | | 0.029 | | |
| REACTOME_INTERFERON_SIGNALING | 152 | -1.62 | | 0.002 | | 0.030 | | |
| REACTOME_MEIOTIC_RECOMBINATION | 80 | -1.61 | | 0.004 | | 0.032 | | |
| REACTOME_INFLUENZA_LIFE_CYCLE | 134 | -1.61 | | 0.000 | | 0.032 | | |
| REACTOME_MITOTIC_G2_G2_M_PHASES | 77 | -1.57 | | 0.007 | | 0.040 | | |
| REACTOME_CLEAVAGE_OF_GROWING_TRANSCRIPT_IN_THE_TERMINATION_REGION | 42 | -1.57 | | 0.016 | | 0.042 | | |
| REACTOME_PHASE_II_CONJUGATION | 61 | -1.55 | | 0.013 | | 0.045 | | |
| REACTOME_MEIOSIS | 108 | -1.54 | | 0.010 | | 0.049 | | |
| REACTOME_MEMBRANE_TRAFFICKING | 124 | -1.53 | | 0.007 | | 0.050 | | |
| REACTOME_UNFOLDED_PROTEIN_RESPONSE | 73 | -1.53 | | 0.007 | | 0.051 | | |
| REACTOME_TRANSLATION | 144 | -1.51 | | 0.002 | | 0.056 | | |
| REACTOME_PROTEIN_FOLDING | 51 | -1.51 | | 0.016 | | 0.056 | | |

Results of gene set enrichment analysis. Size: number of genes in the expression dataset belonging to the pathway; NES: normalized enrichment score; FDR: false discovery rate. Pathways with too long names have been univocally identified posing the corresponding Reactome ID in brackets. Pathways directly cited in the results section are elicited in bold.
