# Supplementary figures and images for: Rewiring of Lipid Metabolism and Storage in Ovarian Cancer Cells after Anti-VEGF Therapy
Source: Cells. 2019 Dec 9;8(12):1601. doi: 10.3390/cells8121601 (PMC6953010; doi:10.3390/cells8121601)

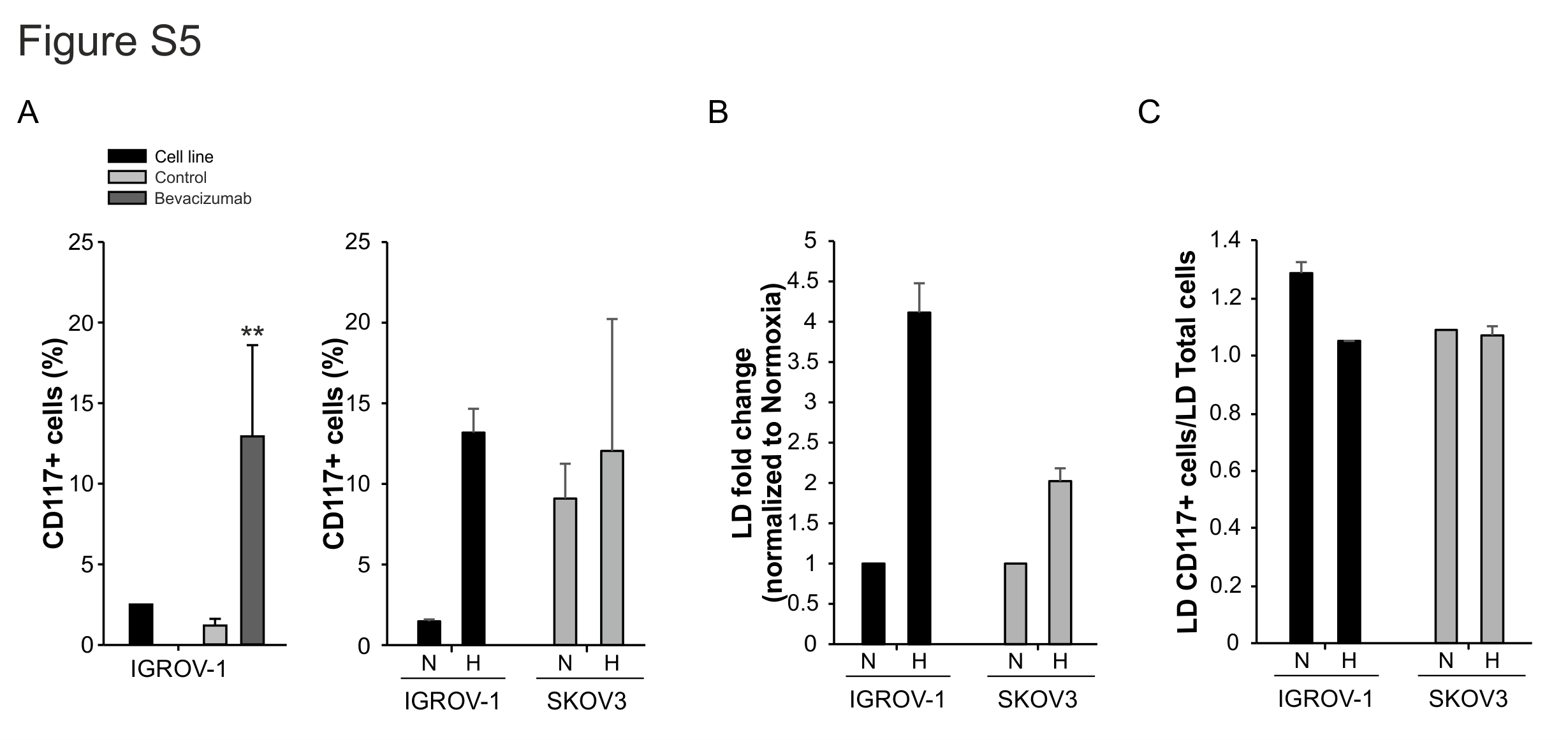

Supplement: Supplementary file 1 [file cells-08-01601-s001.zip › Supplementary Materials 02122019/Figure S5.tif]
